# Rediscovering the chemistry of the *Cunninghamella* species: potential fungi for metabolites and enzymes of biological, industrial, and environmental values

**DOI:** 10.1039/d4ra07187e

**Published:** 2024-12-05

**Authors:** Hosam M. El-Seadawy, Rehan M. El-Shabasy, Ahmed Zayed

**Affiliations:** a Department of Pharmacognosy, College of Pharmacy, Tanta University El-Guish Street (Medical Campus) 31527 Tanta Egypt ahmed.zayed1@pharm.tanta.edu.eg; b Chemistry Department, Faculty of Science, Menofia University 32512 Shebin El-Kom Egypt

## Abstract

Endophytic fungi have a strong affinity for producing the same or comparable compounds to those produced by their hosts. Herein, genetic diversity and environmental adaptation of the *Cunninghamella* species were briefly investigated. The genetic flexibility in *Cunninghamella* represents an evolutionary mechanism that allows them to respond effectively to environmental changes. The current review paid much attention toward the phytochemical screening of *Cunninghamella* sp., revealing the presence of alkaloids, unsaturated sterols, fatty acids, polyphenols, and quinones. The intensive investigations clarified that *Cunninghamella* sp. are distinguished in producing several numbers of fatty acids, in particular polyunsaturated fatty acids (PUFA), in large quantities compared to other metabolites. The study demonstrated the effective role of *Cunninghamella* sp. in forming several bioactive metabolites owing to cytochrome P450 (CYP) that confirm significant value of such species for potential media biotransformation. The comparative investigations revealed that the isolation of flavonoids is yet to be reported, while the number of elucidated alkaloids and steroids is still limited. In contrast, successful results in the biotransformation of these metabolites were verified and showed a high affinity to convert simple substances to more valuable agents by *Cunninghamella*. The biomedical applications of naturally occurring compounds isolated from *Cunninghamella* were well documented; these included their antimicrobial, anti-cancer, anti-inflammatory, anti-Alzheimer, and antiaging properties. The antimicrobial activity was mostly attributed to the fatty acid contents in *Cunninghamella* sp. Moreover, tremendous attention was paid towards the agricultural and industrial usage of chitosan as it is one of the most crucial metabolites involved in wide applications. Chitosan is involved in food preservation for extending life storage period and utilized as biofertilizer, which enhances bacterial disease resistance. In addition, *Cunninghamella* is considered an important enzyme reservoir. Various *Cunninghamella* sp. produce several important enzymes, such as lignin peroxidase, catalase, cellulase, xylanase, laccase, and CYPs, that can be used for remediation, fertilization, preservation and medicinal purposes. Hence, further in-depth investigations are highly recommended to explore new insights into this potential reservoir of a wide spectrum of chemicals for industrial, medicinal, agricultural, and environmental applications.

## Abstract 2

*Cunninghamella* fungi have its unique chemistry that provide potential metabolites applicable for biological, industrial, and environmental fields.

## Graphical abstract

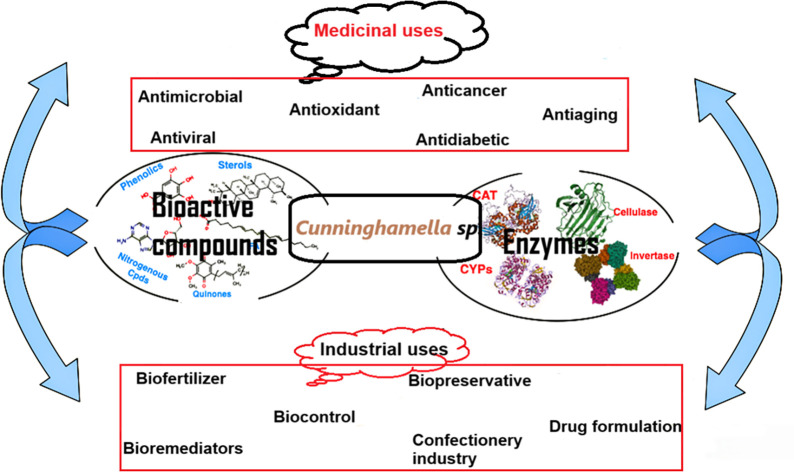

## 1. Introduction

Endophytic fungi are microorganisms that reside within the tissues of the host plant without exhibiting any outward signs of illness.^1^ Several novel endophytic fungi have been recently isolated from different plant species and implemented for several medical and industrial purposes. Endophytes from plants are reported to be a source of several new lead compounds with potential uses in different fields, such as medicine, agriculture, environment, and industry.^2^ Endophytic fungi can produce metabolites like those of their host plants,^2^ such as flavonoids, alkaloids, terpenoids, coumarins, steroids, and lignans.^3^ Previously isolated fungal metabolites have verified that the diversity in biological activities encompasses antioxidant, anticancer, antimicrobial, anti-respiratory syncytial, antiproliferative and antibacterial functions.^4,5^^.^ For instance, a myriad of biologically active components, including terpenoids, alkaloids and flavonoids, were isolated from the *Phyllosticta* sp. fungus, which showed potent antioxidant activity.^6^ In addition, a recent phytochemical study successfully utilized the endophytic fungi *Coniolariella hispanica*, *Penicillium canescens*, *Paraphoma radicina* and *P. murcianum* as precursors for producing cryptotanshinone as a terpene compound that considered the principal metabolite like the host *Salvia abrotanoides* plant.^7^ An additional distinctive bioactive agent was identified with 3-hydroxy-4-(hydroxy(4-hydroxyphenyl) methyl) dihydrofuran-2-on from *Fusarium verticillioides*, proving its antibacterial activity.^8^ Mellein and *β*-retinaldehyde were also isolated from the endophytic fungus *Botryosphaeria fabicerciana*, of which its potential efficacy as antioxidant and antimicrobial was revealed.^9^ Beside its effective role in biological activity, endophytic fungi also enhanced plant growth and development *via* the secretion of different enzymes involved in the biotechnological and industrial sectors.^10^

Taking into consideration the potential attention paid towards endophytic fungi, Mucorales is the largest order.^11^ This order belongs to the phylum Zygomycota, subphylum Mucoromycotina^11^ that comprises 15 families, 57 genera and 334 species.^12^*Cunninghamella* (family *Cunninghamellaceae*) was the most predominated genus belonging to Mucorales order and is one of the most potential fungi which has been investigated deeply.^13^*Cunninghamella* was first established by Matruchot in 1903 upon collection of *C. africana* in the French Sudan, which would later be known as *C. echinulata*.^14^*Cunninghamella* sp. has a strong ability to produce sporophores with uni-spored sporangia that are pedicellate on the vesicle surface and coated in spines. The sporophores have an uneven, pseudo-vertical, or verticillate branching shape.^14^ Furthermore, the majority of *Cunninghamella* sp. are saprobes, which are frequently founded in soil, stored grains, and other organic substrates.^15^ On the other hand, to identify additional species belonging to *Cunninghamella*, physical properties can be used, such as the colony color, texture, pattern of sporophore branching, vesicle form and size, sporangia shape and size, and the presence or absence, and length of spines in the sporangia.^14^ Based on the phylogenetic analyses and morphological characters, there are about 17 species of *Cunninghamella* that have been identified.^16^ In immunocompromised patients, such as those who have undergone hematopoietic stem cell transplants or hematological malignancies, some *Cunninghamella* sp. such as *C. bertholletiae*,^17^*C. blakesleeana*,^18^*C. echinulata*,^19^*C. elegans*^15^ and *C. arunalokei*,^20^ can cause mucormycosis, an angioinvasive illness that primarily manifests as pulmonary and disseminated infections.^21,22^ Different *Cunninghamella* sp. can produce a variety of secondary metabolites with promising medical and industrial values.^23^ Intensive and recent investigations have triggered attention toward the successful utilization of the *Cunninghamella* species in drug fabrication, owing to the presence of CYP-450 monooxygenase systems that are analogous to those in mammals.^24^ In addition, *Cunninghamella* is regarded as a major source of a wide range of enzymes that can be employed in industry, bioremediation, and biotechnological features, including the biotransformation of various pharmaceutically significant substances like steroids and terpenoids,^25^ because it could convert the substrate into highly active compounds.^26^

The current review aims to highlight and extend the potential of the genus *Cunninghamella* in providing different classes of bioactive secondary metabolites. Interestingly, there is still limitation for direct isolation of lead compounds from *Cunninghamella* sp. However, most of the intensive investigations have paid more attention to the biotransformation process. *Cunninghamella* sp. has a strong capacity to use fewer effective compounds as the initial precursors, and convert them into structurally valuable molecules that are widely applied in drugs. This is attributed to the significant genes and enzymes included in *Cunninghamella* sp. that play an important role in biotransformation, and could be involved in industrial and biotechnological applications. In this review, a broad range of studies have been demonstrated for gaining comprehensive information about *Cunninghamella*.

## 2. Genetic diversity and functional capabilities of the *Cunninghamella* species

The genus *Cunninghamella* comprises several key species, including *C. elegans*, *C. bertholletiae*, and *C. echinulata*. These fungi exhibit significant genetic diversity, which influences their biochemical functions and potential applications in various fields.

The CYP-450 genes are essential to the metabolic capabilities of *Cunninghamella* sp. For instance, the CYP5208A3 gene in *C. elegans* plays a crucial role in metabolizing a wide variety of chemical compounds.^27^ This gene contains coding regions that determine the protein's sequence, as well as regulatory elements such as promoters and enhancers, which control gene expression in response to environmental stimuli.^28^ The interaction between gene expression and other metabolic pathways enhances the enzyme's ability to chemical processing, demonstrating the organism's adaptability and potential applications, such as pollutant treatment and the development of new chemicals.^29,30^ Environmental adaptation is crucial for the survival and persistence of *Cunninghamella* sp. in diverse environments. These fungi possess a unique ability to adapt to various environmental conditions, such as temperature, humidity levels, and the presence of pollutants due to their genetic diversity, particularly CYP genes. This genetic diversity enables the fungi to process a wide range of chemical compounds, making them resilient and capable of adapting to environmental changes.^31,32^ For example, exposure to toxic compounds can trigger an increase in the expression of specific CYP genes, enhancing the fungi's ability to neutralize these compounds.^33,34^ The genetic adaptation can directly impact the survival of fungi in polluted or changing environments.^35^ Previous investigations suggested that these fungi possess advanced genetic modification capabilities, enhancing them to develop immediate and long-term responses against environmental changes.^28^ Studies revealed significant variation in the number of CYP genes amongst different *Cunninghamella* sp.; for example, *C. bertholletiae* possess 69 CYP genes, whereas *C. elegans* have only 32.^36^ The variation in gene numbers suggested a greater biochemical transformation capacity in *Cunninghamella*, influencing its ability to adapt to various chemical environments.^26,37^ Strains with extensive CYP gene families, like those found in *C. bertholletiae*, can efficiently degrade complex pollutants, thereby supporting their use in biotechnological and environmental contexts.^38^ This adaptability highlights the importance of *Cunninghamella* sp. in addressing pollution and chemical processing challenges.^31,32^

*Cunninghamella* sp. exhibit a remarkable ability to adapt to environmental conditions. This adaptability is attributed to the fungi's ability to modulate gene expression in response to environmental conditions. For instance, certain strains of *Cunninghamella* can modify their genetic makeup to develop resistance against chemical pollutants or environmental changes, giving them a competitive advantage in complex environments. Their ability to produce enzymes that can degrade environmental pollutants or transform industrial chemicals into less harmful substances is particularly notable. For example, *C. elegans* has been studied for its role in the biotransformation of pharmaceuticals, which highlights its potential in developing environmentally friendly waste treatment processes.

## 3. Previously isolated metabolites from *Cunninghamella* species

*Cunninghamella* sp. are a valuable source for a plethora of naturally occurring compounds with promising efficiency in biological, industrial, agricultural, and environmental applications. Relevant and recent studies have described the isolation of different secondary metabolites like fatty acids, sterols, phenolic acids, quinones, polysaccharides and nitrogenous compounds from *Cunninghamella* sp. It is worth mentioning that few compounds have been isolated from *Cunninghamella* sp., compared to the high numbers of biotransformed metabolites, as presented in Table 1. *Cunninghamella* has a strong ability to metabolize numerous chemical agents in regio- and stereo-selective strategies, according to the phase I (oxidative) and phase II (conjugative) biotransformation mechanisms. Concurrently, *Cunninghamella* is considered as one of the significant and distinguished biotechnological techniques regarding biotransformation.^39^

**Table:** Screening of the bio-transformed compounds forming more effective flavonoids by the *Cunninghamella* species

*Cunninghamella* species	Precursors	Producing metabolites	Spectroscopic analysis	Application	Ref.
*C. elegans*	Coumarins	3, 4-Dihydrocoumarin	NA^a^	Cytotoxic	46
		Umbelliferone			
	Dicoumarol	4-Hydroxycoumarin			
*C. blakesleeana*	Icariin, epimedin C, epimedoside A, epimedin A, epimedin B	Icariside II	NMR	Anti-osteoporosis	43
		2-*O*-Rhamnosylikarisoside II			
		Epimedoside b			
		Baohuoside VII			
		Sagittatoside B			
*C. blakesleeana*	Norkurarinone	Kurarinone	NA^a^	Cytotoxic	44
		4′′,5′′-Dihydroxykurarinone			
		6′′-Hydroxyl-2′-methoxyl-norkurarinone 7-*O-β*-d-glucoside			
		6′′-Hydroxylnorkurarinone 4′-*O-β*-d-glucoside			
		7-Methoxyl-4′′,5′′-dihydroxynorkurarinone			
*C. echinulata*	Kurarinone	6′′-Hydroxykurarinone	NA^a^	Cytotoxicity	45
		4′′,5′′,8′′-Trihydroxynorkurarinone			
		Norkurarinone			
		Kurarinone 7-*O-β*-d-glucoside			
*C. blakesleeana*	Silybin	Silybin-7-sulfate	NMR	Antioxidant	47
		2,3-Dehydrosilybin-7-sulfate			

a NA: not available.

### 3.1. Flavonoids

**Fig. 1:**
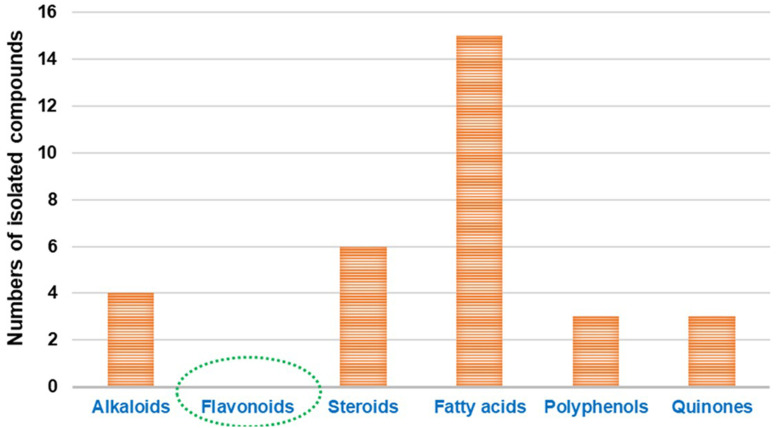
Comparison between the previously identified metabolites isolated from the *Cunninghamella* species.

The bioactive flavonoids represent the most crucial phytochemicals isolated from plants, fungi and other natural sources, revealing a wide range of biological benefits for the human body. The complex structures of flavonoids make their extraction from plants difficult, and there are additional problems associated with the chemical synthesis due to the utilization of several toxic solvents. Microbial production is considered a preferable method for the isolation of these metabolites for industrial applications, as it is a more economical, sustainable and eco-friendly approach.^40^ However, with the advances in isolation techniques, microbial production is still limited on a laboratory scale. Furthermore, its enhancement and large-scale fabrication remain a great challenge. Interestingly, despite their potential activity and low toxicity, none of these compounds have yet been isolated directly from *Cunninghamella* sp., as introduced in Fig. 1. Clearly, based on the literature review, most reported studies have discussed the vital role of *Cunninghamella* in the successful biotransformation of less effective compounds used as the substrate and converted them into more valuable flavonoids and flavonoid derivatives to maximize their impact in drug discovery (Table 1).^41^

**Fig. 2:**
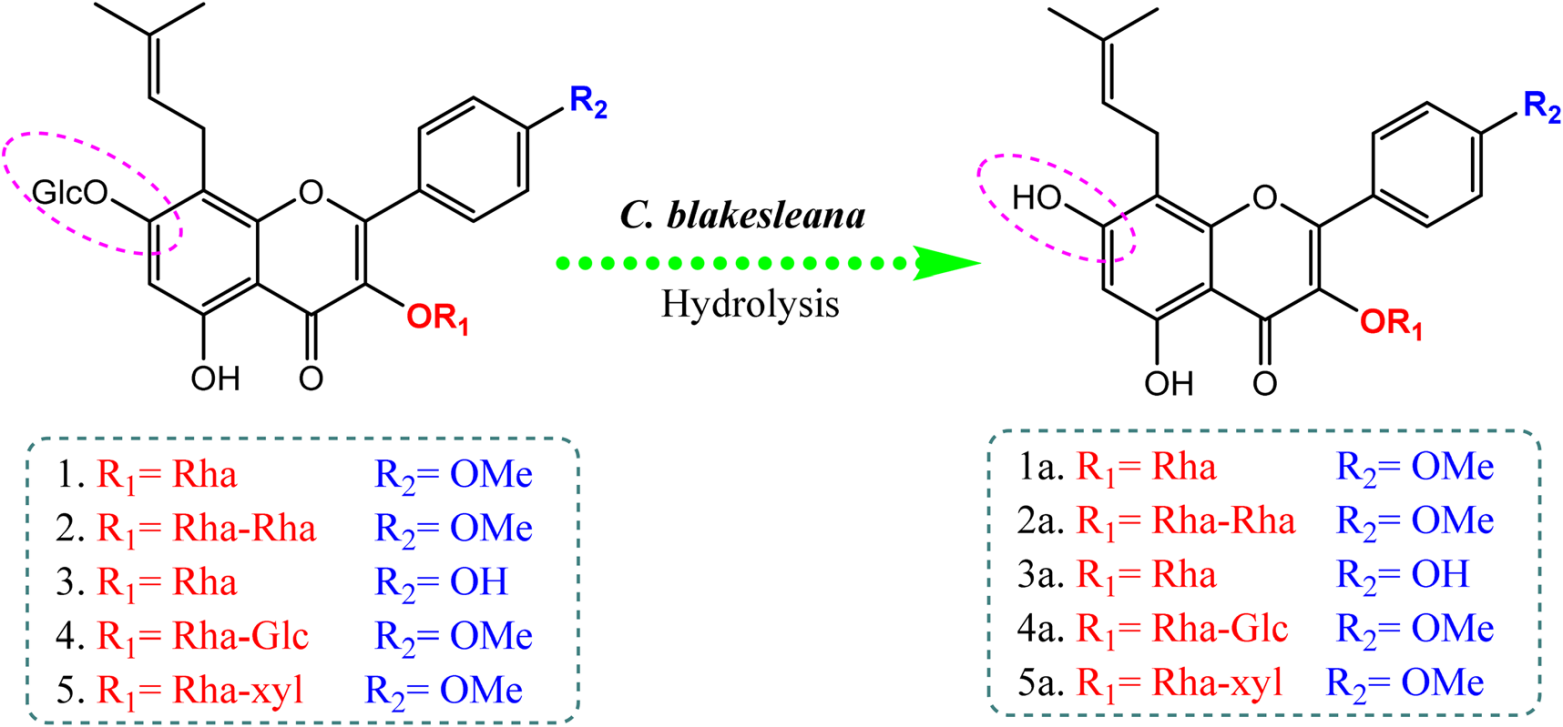
Bio-transformation of flavonoids to rare flavonoid glycosides by *Cunninghamella blakesleeana via* hydrolysis reaction.^43^

**Fig. 3:**
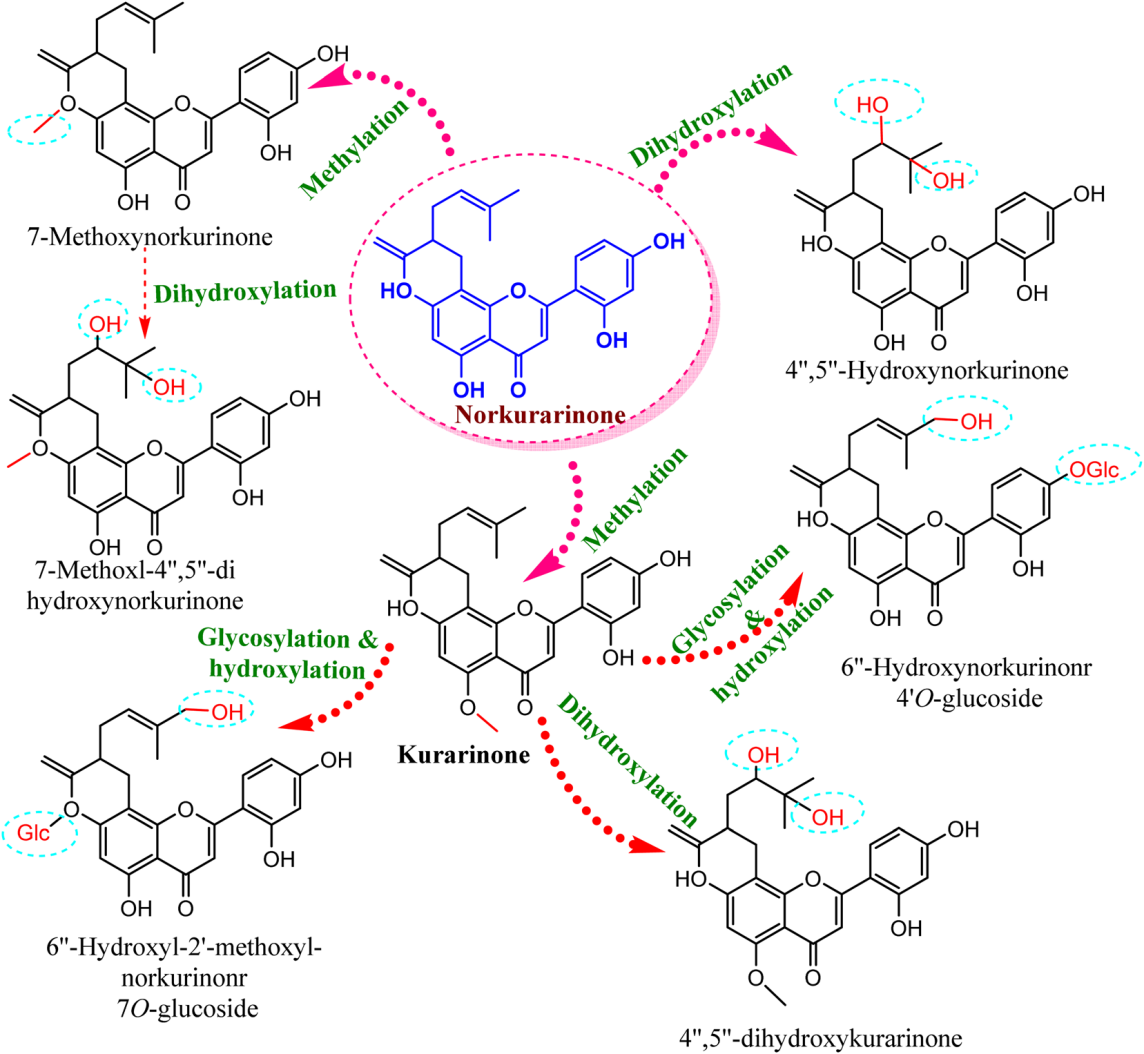
Biotransformation reaction of norkurarinone by *Cunninghamella blakesleeana* and *C. echinulata*.

Compared to the isolated flavonoids, several numbers of bio-transformed flavonoids have been facilely produced by *Cunninghamella*. Noticeably, the basic reactions associated with microbial biotransformation included glycosylation/deglycosylation, carbonyl reduction, hydroxylation/dehydroxylation, *O*-methylation/*O*-demethylation, cyclization, hydrogenation/dehydrogenation, sulfation and C ring cleavage of the benzo-*γ*-pyrone system.^42^*Aspergillus*, *Penicillium* and *Cunninghamella* sp. are the most prevalent genera in flavonoid biotransformation, and they are distinguished in their ability to perform nearly the entire reactions with significant yields.^42^ For instance, *C. blakesleeana* has been incorporated in the biotransformation of the principal flavonoid glycoside shown in Fig. 2, and is isolated from the herb *epimedii* for producing a number of rare flavonoids with excellent yield (<95%).^43^*Cunninghamella* boosts the potential selectivity of C-7 hydrolysis to form a number of unexpected flavonoid glycosides, including icariside II (95.1%), 2-*O*-rhamnosylikarisoside II (97.7%), epimedoside b (93.7%), baohuoside VII (95.8%) and sagittatoside B (96.4%), as presented in Fig. 2.^43^*C. blakesleeana* was also utilized in the biotransformation of norkurarinone to kurarinone, 4′′,5′′ dihydroxynorkurarinone, 7-methoxyl-norkurarinone, 6′′-hydroxyl-2′-methoxyl-norkurarinone-7-*O-β*-d-glucoside, 6′′-hydroxylnorkurarinone-4′-*O-β*-d-glucoside, 4′′,5′′-dihydroxykurarinone and 7-methoxyl-4′′,5′′-dihydroxynorkurarinone, as shown in Fig. 3.^44^ Furthermore, kurarinone was biotransferred to afford flavonoid derivatives using *C. echinulata via* hydroxylation, dihydroxylation on the C4′′ = C5′′, *O*-methylation and glycosylation reactions^45^ (Fig. 3) (Table 1).

**Fig. 4:**
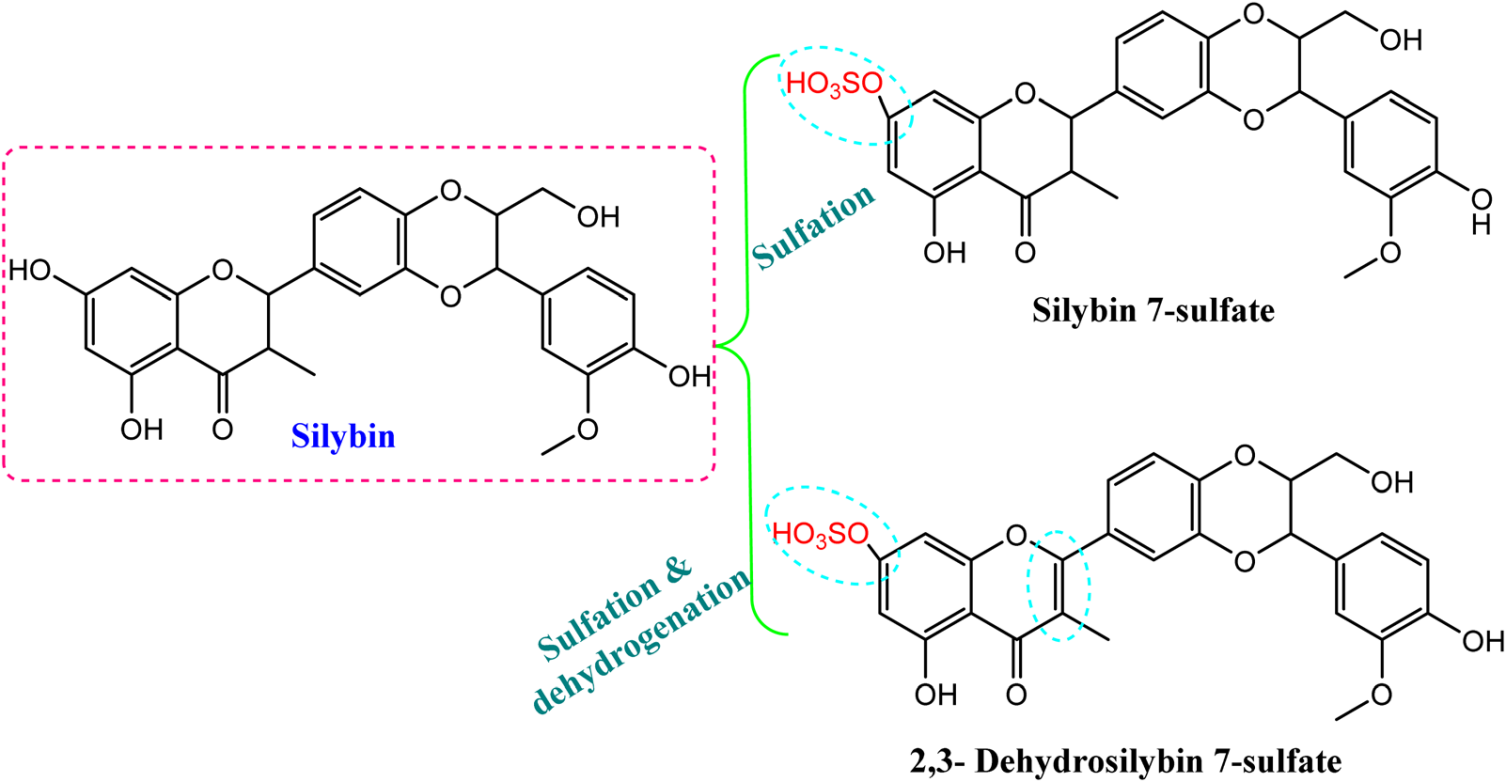
Sulfation and dehydrogenation of the flavonolignan, silybin, by *Cunninghamella* sp.

Another study has introduced the efficient metabolized coumarin into 3,4-dihydrocoumarin, umbelliferone and *trans*-cinnamic acid using C. *elegans* NRRL 1392 and dicoumarol, which was transformed into 4-hydroxycoumarin. The produced compounds were characterized by different spectroscopic techniques (*e.g.*, NMR, mass spectrometry) and showed cytotoxic activity^46^ (Table 1). In addition, microbial biotransformation of the major flavolignan founded in milk thistle, silybin, by *C. blakesleeana* resulted in isolation of 2,3-dehydrosilybin 7-sulfate and silybin 7-sulfate, as shown in Fig. 4.^47^ A sulfation reaction was induced on C-7 of silybin, which potentially reduced the DPPH free radical scavenging activity. Meanwhile, dehydrogenation at C2 = C3 produced 2,3-dehydrosilybin 7-sulfate, which significantly enhanced the antioxidant activity.^47^

As a result, it was clearly observed that *Cunninghamella* sp. is distinguished for producing diverse and rare flavonoids *via* the biotransformation process, in comparison to the isolated metabolites.

### 3.2. Alkaloids

A number of recent studies have focused on the isolation of newly discovered alkaloids from plants. However, very few of these molecules have been isolated from *Cunninghamella* sp. For instance, Inoue *et al.* discussed the isolation of a new tricyclic alkaloid named ICM020 from *Cunninghamella* sp. F-1490.^48^ The structure of this compound was determined to be (3*S*, 10a*R*)-3,4a-dihydroxy-2,3,4,4a-tetrahydro-2*H*-pyrano[3,2-*b*]benzo[*e*]morpholine-9-carboxylic acid using various spectroscopic techniques.^49^ This compound was found to have osteoclastogenic inhibitory activity. Using the parathyroid hormone related peptide (PTHrP) test method and tartrate-restricted acid phosphatase (TRAP)-positive multinucleated cells formation assay method, a significant inhibitory impact was demonstrated against the development of osteoclasts in mouse bone marrow cells with an IC_50_ value of 0.78 μg mL^−1^. Furthermore, this compound showed weak cytotoxicity against bone marrow cell lines using the trypan blue exclusion assay, which suggested that its cytotoxicity was not a factor in the prevention of osteoclastogenesis.^48,50^ Some nucleosides and nucleotides were also reported from *Cunninghamella* sp., such as adenosine, uridine and uracil, which were isolated and fully characterized from *C. elegans*.^51^ Adenosine is known as an antiarrhythmic drug, and is found commercially as Adenocard®. The identified metabolites were investigated for their wound healing efficiency against pigs.^51^ To the best of our knowledge, this is the comprehensive series of isolated alkaloids derived from *Cunninghamella* sp., and future research may reveal new bioactive molecules.

**Fig. 5:**
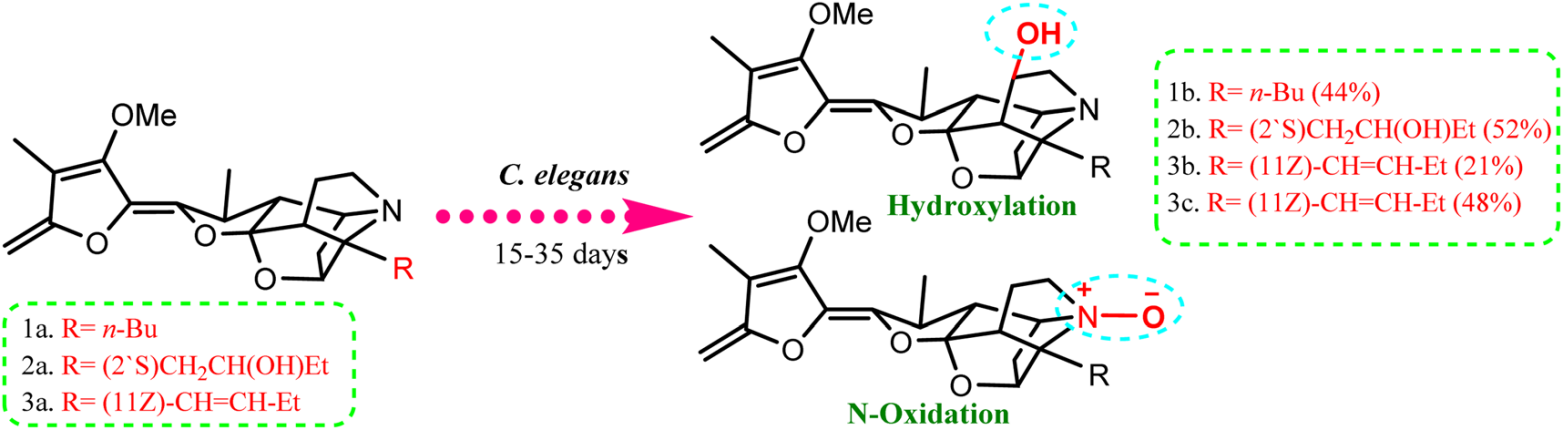
Oxidation and hydroxylation biotransformation of stemofoline alkaloids through fermentation by *Cunninghamella elegans* TIST 3370.

**Fig. 6:**
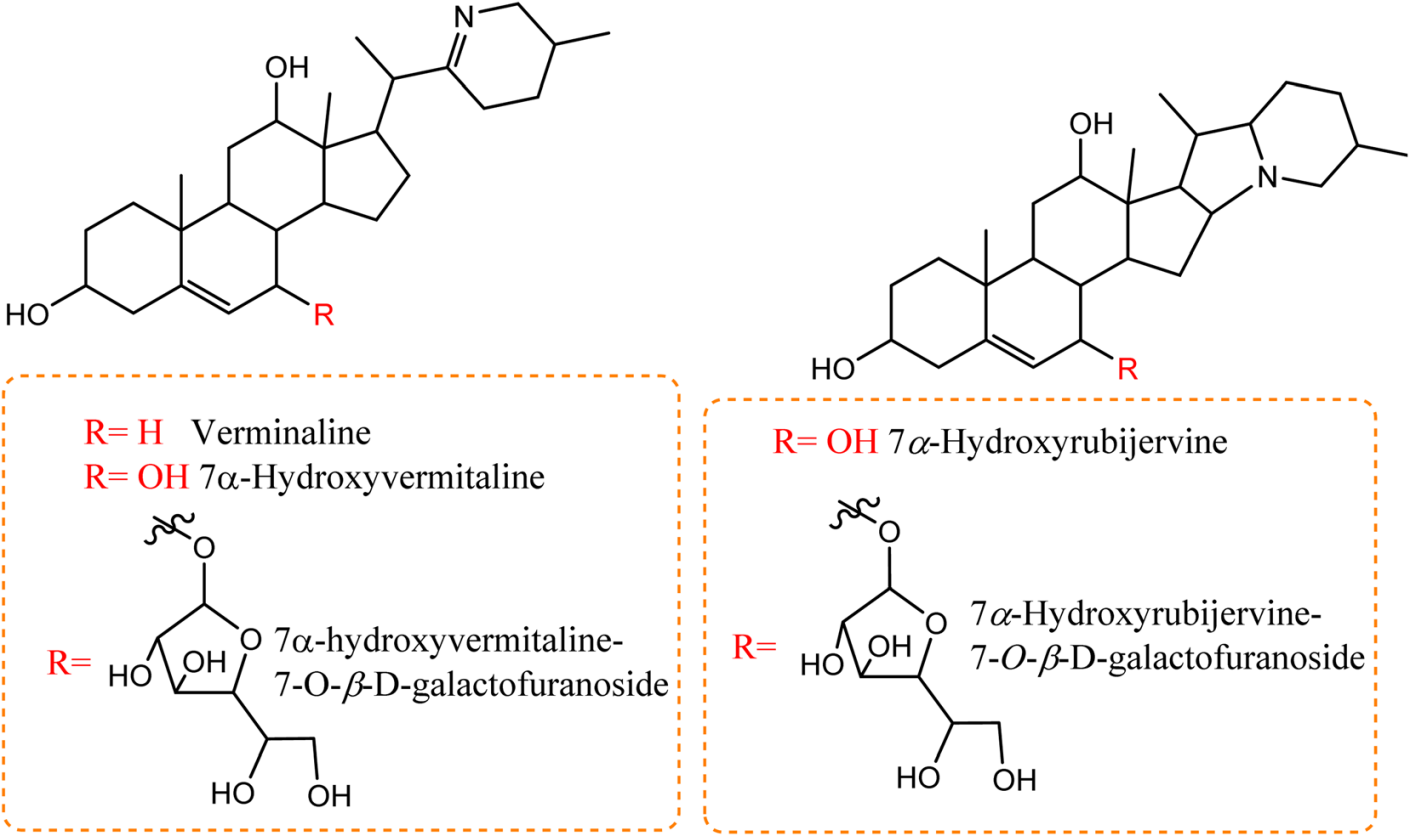
New alkaloid derivatives being transformed from verminaline by *Cunninghamella echinulata*.

Compared to the isolated alkaloids from *Cunninghamella*, biotransformation has become the leading strategy for producing several compounds that have significant biodiversity. For example, Chalom *et al.* reported on the oxidative transformation of stemofoline during fermentation with *C. elegans* TISTR. The study resulted in three bioactive alkaloid derivatives (shown in Fig. 5) that displayed potential inhibition against acetylcholinesterase with (IC_50_ = 11.01 ± 1.49 mM) compared to the precursor (IC_50_ = 45.1 ± 5.46 mM).^52^ Lü *et al.* have also investigated the biotransformation of vermitaline *via C. echinulata* (ACCC 30369), producing new alkaloid derivatives that were characterized by NMR and mass spectra.^53^ The biotransformed compounds were identified as 7α-hydroxyvermitaline-7-*O-β*-d-galactofuranoside, 7*α*-hydroxyrubijervine-7-*O-β*-d-galactofuranoside, and 7*α*-hydroxyrubijervine, 7*α*-hydroxyvermitaline, as shown in Fig. 6.^53^

Based on the literature, it was clearly noted that until now, few reports have discussed the isolation and biotransformation of alkaloids by *Cunninghamella* sp., despite their potential efficacy in drugs (Table 2). A huge gap is still being observed between *Cunninghamella*-derived alkaloids compared to the estimated number of plant-derived alkaloids represented by <60% of all potential drugs.^54^ Hence, further investigations regarding *Cunninghamella*-derived alkaloids are highly recommended, which could be a fruitful source for new bioactive agents for efficient drugs.

**Table:** Bio-transformed metabolites for producing alkaloids by *Cunninghamella* sp.

*Cunninghamella* species	Precursors	Producing metabolites	Spectroscopic analysis	Application	Ref.
*C. elegans* TISTR 3370	Stemofoline	(6*R*)-Hydroxystemofoline	NMR	Inhibitor against acetylcholinesterase	52
		(2′*S*)-Hydroxystemofoline			
		(11*Z*)-1′,2′-Didehydrostemofoline & 1′,2′-didehydrostemofoline-*N*-oxide			
*C. echinulata* (ACCC 30369)	Vermitaline	7a-Hydroxyrubijervine	MS, NMR	ND^a^	53
		7α-Hydroxyrubijervine-7-*O-β*-d-galactofuranoside			
		7*α*-Hydroxyvermitaline			
		7*α*-Hydroxyrubijervine-7-*O-β*-d-galactofuranoside			
		6*β*,17*β*-Dihydroxy-7*α*,17*α*-dimethylestr-4-en-3-one			
		6*β*,10*β*,17β-Trihydroxy-7*α*,17*α*-dimethylestr-4-en-3-one			

a ND: not determined.

### 3.3. Fatty acids

Several *Cunninghamella* sp., like *C. elegans*, *C. echinulata* and *C. bainieri*, have shown a potential affinity toward the fabrication of a plethora of lipid compounds.^55^ Polyunsaturated fatty acids (PUFA) can be accumulated in significant quantities by *Cunninghamella*, such as *γ*-linoleic acid (GLA).^56^ Different chromatographic and spectroscopic methods were used to determine the PUFA profile of various *Cunninghamella* sp. One of the main PUFA in *Cunninghamella* is GLA, which represents 21.7% and 21.1% of the total lipid content in *C. echinulata* and *C. blakesleeana*, respectively.^57–59^ GLA plays a crucial role in brain function and normal growth with developments.^60^ An additional investigation recently analyzed the fatty acid (FA) contents in *C. echinulata* using GC-MS. Experimentally, the chemical profile analysis demonstrated the variation percentages of several FAs; saturated FAs comprised 44.49%, monounsaturated FAs comprised 19.91%, and PUFAs comprised 35.61%. Linoleic acid recorded the highest value at 13.79%, followed by stearic (12.78%) and then oleic acid (12.50%), while GLA yielded a suitable percent (8.02%). Additional types of PUFAs appeared, including eicosapentaenoic acid, arachidonic acid, and docosahexaenoic acid.^61^ The production yield of FAs could be increased through optimization of the cultivation conditions. A comparative study after several trials revealed that lipids (1.43 g L^−1^) could be accumulated in *Cunninghamella* sp. under the conditions of 5 g peptone and 20 g sucrose for 9 days.^62^ Under the optimum conditions after 6 days, the majority of the produced FA was saturated. Meanwhile, the PUFAs were the minor component, including palmitic acid, followed by oleic acid and GLA. In contrast, the FA profile was wholly changed under different conditions (9 days, followed by 3 days at 15 °C) such that oleic acid was predominant with increasing percent of GLA from 1.14 to 3.22%.^62^

In the continued search for isolated FAs from *Cunninghamella* sp., Salicorn 5 was also employed in the isolation of a number of PUFAs, like linoleic acid, GLA, oleic acid and other lipids.^63^ Although the amount of lipids produced by Salicorn 5 (*Cunninghamella* sp.) was relatively small compared to those formed in vegetables (*e.g.*, rapeseed oil at 35–40%), the short generation time and high growth rate of fungi make their continued investigation worthwhile.^63^ Additional reports have investigated the isolation and identification of stearic, palmitic and oleic acids from *C. blakesleeana* and *C. elegans* extracts using column chromatography.^51,64,65^ Gas chromatography (GC) was also successfully employed for the characterization of several FAs in various quantities from *C. blakesleeana* biomass. The major FA was stearic acid (74.61%), followed by palmitic acid (10.35%), whereas the lowest percent was characterized by arachidic acid.^64^ Consequently, based on the relevant results, *Cunninghamella* sp. could be used as a commercial source for these types of secondary metabolites.

An in-depth investigation showed that *Cunninghamella* is superior in the isolation of several FAs, while the biotransformed compounds associated with this class have yet to be reported. Finally, it is worth mentioning that essential FAs cannot be synthesized by humans, and it must be obtained from diets. Analysis of the FA composition showed that PUFAs represented 87.03% of the total FAs, comprising oleic acid (35.57%), linoleic acid (21.58%), palmitoleic acid (16.31%), and linolenic acid (13.28%), while eicosenoic acid, stearic acid, myristic acid, and arachidic acid were found in much lower amounts.^63^ A relevant investigation revealed that the lipid compositions were comparable to those of the edible oils and fats; hence, *Cunninghamella* sp. could be successfully applied as an important source of edible oils.^63^

### 3.4. Steroids

Steroids have emerged in living organisms with diverse and potential activity. Phytosterol compounds were reported from various *Cunninghamella* sp., including *β*-sitosterol and α-amyrin from *C. elegans* and *C. blakesleeana*, in addition to stigmasterol from *Cunninghamella* sp. In general, these compounds are commonly known in the plant kingdom as having a wide range of medical purposes.^55^ For instance, the leaves of *Odontonema strictum* are rich in *β*-sitosterol and stigmasterol valued at 60% and 40%, respectively.^66^*α*-Amyrin has emerged in several plant species with potential activities (*e.g.*, anti-inflammatory, antitumor, hepatoprotective and anxiolytic). However, the accurate determination of this compound in plant sources is still limited due to problems associated with efficient isolation, identification and quantification. Besides that, the isolation of such compounds for examination against diseases has not been specifically established.^67^ As a result, *Cunninghamella* could be an alternative source for such compounds and a suitable candidate for discovering new bioactive agents. In addition, a previous study has reported that ergosterol, stigmasta-7,22-diene-3*β*,5*α*,6*α*-triol and stigmasterol have been isolated from Salicorn 5 (*Cunninghamella* sp.).^63^ These compounds were also produced from halophyte *Salicornia bigelovii*,^68^ explaining that endophytic fungi have a potential affinity to create the same chemical compounds or comparable ones produced by their hosts.

**Fig. 7:**
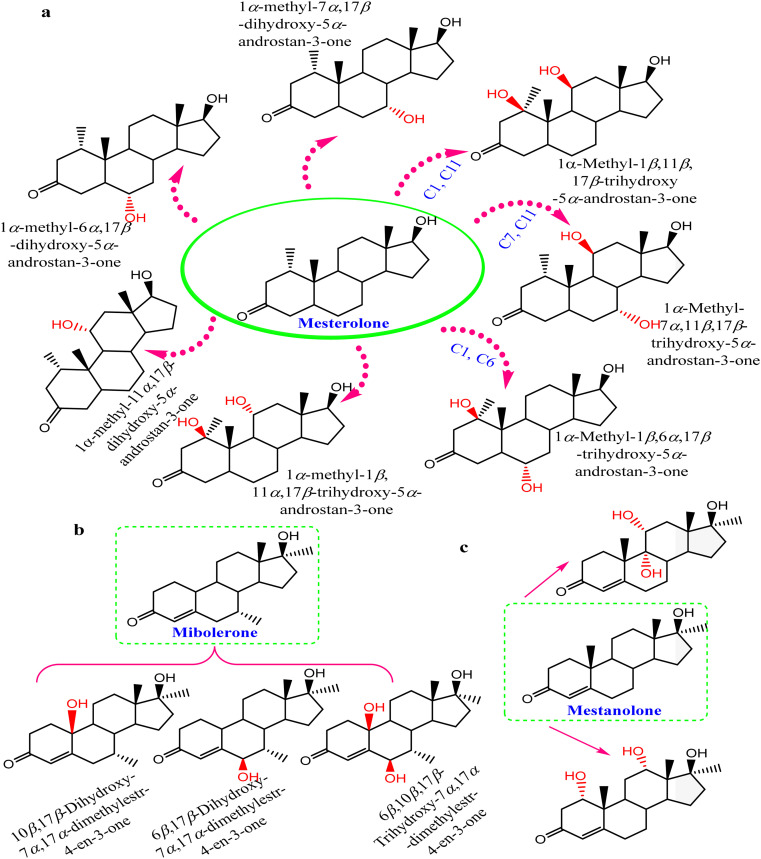
Steroidal hydroxylated biotransformation of (a) mesterolone, (b) mibolerone, and (c) mestanolone with *C. blakesleeana* and *C. echinulata*.

**Fig. 8:**
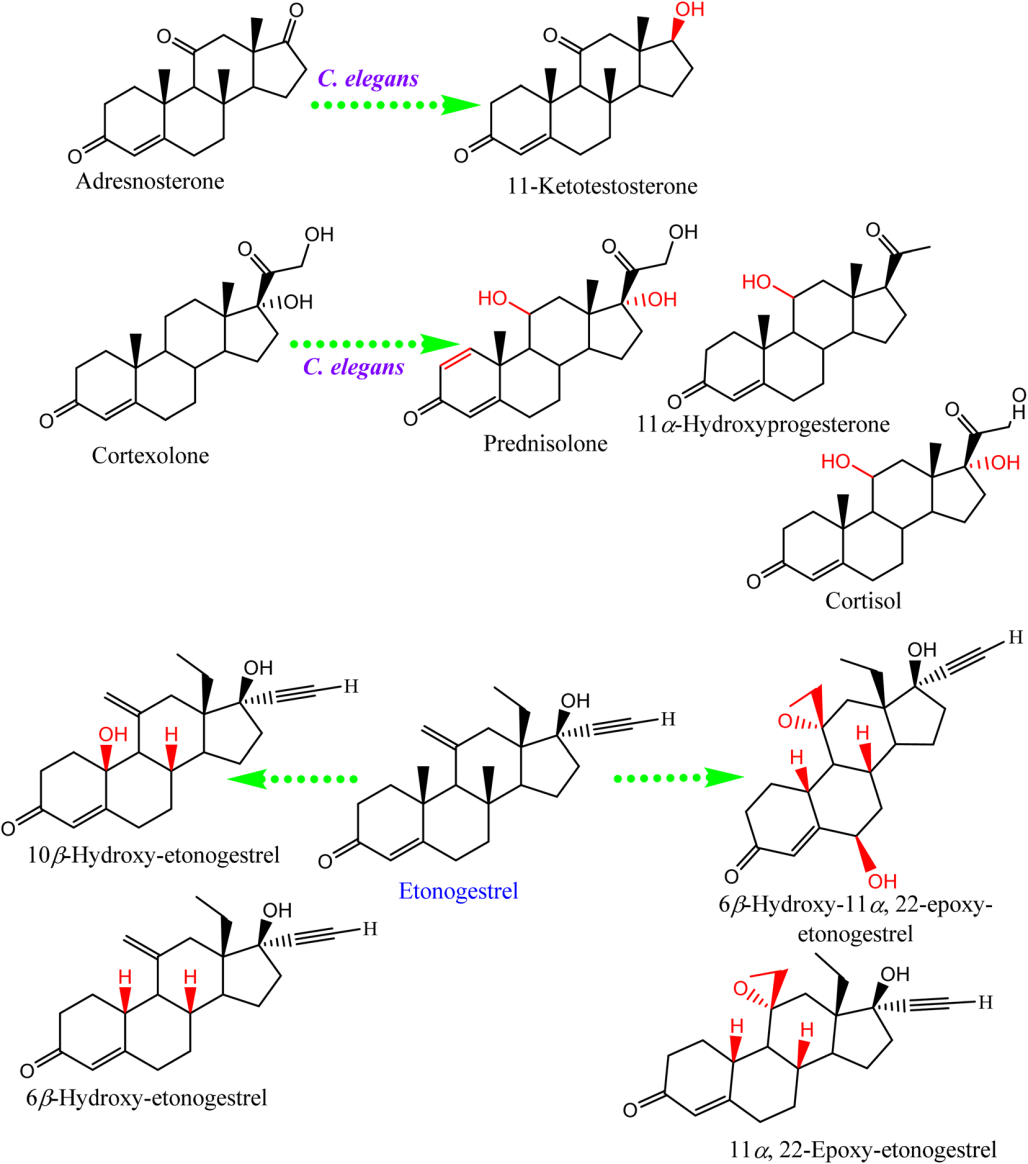
Examples of steroidal biotransformation by *Cunninghamella* sp.

Conversely, biotransformation action plays a vital role in revealing the biodiversity in steroid production by *Cunninghamella* (Table 3). For instance, *C. echinulata* was involved in the isolation of ergosterol, besides two novel adipate esters from fusidic acid.^69^ The species was also used in a formylation reaction to obtain a unique fusidic acid derivative identified as 3-*O*-formyl-27-hydroxyfusidic acid.^70^ The chemical structures were elucidated by intensive spectroscopic methods like 1D, 2D-NMR and HRESIMS. *In silico* studies induced a significant agonist/antagonist effect through binding to the μ opioid receptor and antidiabetic activity *via* aldose reductase inhibitory action.^69^ It was observed that fusidic acid in mammals can be metabolized *via* C-3 or C-27 oxidation and glucuronide conjugation. Compared to mammals, microbes used C-3 and C-6 oxidation, C-6 and C-7 hydroxylation, and deacetylation of C-16, and then spontaneous lactone formation.^71,72^ Interestingly, modification on the side chain of fusidic acid rarely occurred. However, among several organisms, *C. echinulata* was the most efficient fungi in biotransformation, causing oxidation successfully at C-26 and C-27.^73^ Furthermore, a number of bioactive stereoselective derivatives were created such as fermentation of mesterolone by *C. blakesleeana*, producing a number of stereoselective steroids (as shown in Fig. 7a), which were investigated against different activities like anticancer, phosphodiesterase-5 enzymes, and oxidative burst.^74^ Moreover, three additional new steroids were obtained from the biotransformed androgenic steroid mibolerone with *C. blakesleeana* and *C. echinulata*.^75^*Cunninghamella* sp. showed high capacity to catalyze the hydroxylation at the allylic positions of C-1, C-6, C-10, C-11, and C-20. C-6, C-10, and C-11 were the sites for β-hydroxylation, whereas α-hydroxylation occurred at C-1, as shown in Fig. 7b. The produced compounds were investigated against different activities, including *β*-glucuronidase inhibitory, anticancer and leishmanicidal activity.^75^ By the same way, mestanolone was also hydroxylated and transformed by *C. blakesleeana* to afford new steroidal derivatives, as shown in Fig. 7b. A number of steroidal derivatives were fabricated *via* microbial transformation of etonogestrel utilizing *C. echinulata* and *C. blakesleeana*, which showed cytotoxic activity.^76^ The transformed molecules were biosynthesized through epoxidation and hydroxylation at C-6, C-10, and C-15, whereas the epoxy ring was formed between the C-11 and C-22 positions (Fig. 8).^76^ Moreover, the biotransformation of adrenosterone and cortexolone resulted in the production of new derivatives with *C. elegans via* hydroxylation, as presented in Fig. 8.^77,78^ Notably, from the relevant and aforementioned studies, it was observed that steroidal biotransformation basically occurred *via* hydroxylation at different positions of the steroid skeleton.

**Table:** Steroidal biotransformed products by *Cunninghamella* species

*Cunninghamella* species	Precursors	Producing metabolites	Spectroscopic analysis	Application	Ref.
*C. blakesleeana*	Mesterolone	1*α*-Methyl-1*β*,11*β*,17*β*-trihydroxy-5*α*-androstan-3-one	1D, 2D-NMR, HRESI-MS	Anti-cancer, phosphodiesterase-5 enzymes, oxidative burst	74
		1*α*-Methyl-7*α*,11*β*,17*β*-trihydroxy-5*α*-androstan-3-one			
		1*α*-Methyl-1*β*,6*α*,17*β*-trihydroxy-5*α*-androstan-3-one			
		1*α*-Methyl-1*β*,11*α*,17*β*-trihydroxy-5*α*-androstan-3-one			
		1*α*-Methyl-11*α*,17*β*-dihydroxy-5*α*-androstan-3-one			
		1*α*-Methyl-6*α*,17*β*-dihydroxy-5*α*-androstan-3-one			
		1*α*-Methyl-7*α*,17*β*-dihydroxy-5*α*-androstan-3-one			
*C. blakesleeana* and *C. echinulata*	Mibolerone	10*β*,17*β*-Dihydroxy-7*α*,17*α*-dimethylestr-4-en-3-one	1D & 2D-NMR	*β*-Glucuronidase inhibitory, anticancer and leishmanicidal activity	75
		6*β*,17*β*-Dihydroxy-7*α*,17*α*-dimethylestr-4-en-3-one			
		6*β*,10*β*,17*β*-Trihydroxy-7*α*,17*α*-dimethylestr-4-en-3-one			
*C. blakesleeana*	Mestanolone	9*α*,11*β*,17*β*-Trihydroxy-17*α*-methyl-5*α*-androstan-3-one	MS, ^1^H-, ^13^C and 2D-NMR, X-ray diffraction	Anticancer, immunomodulatory	79
		1*β*,11*α*,17*β*-Trihydroxy-17*α*-methyl-5*α*-androstan-3-one			
*C. blakesleeana* & *C. echinulata*	Etonogestrel	6*β*-Hydroxy-11,22-Epoxy-etonogestrel	HREI-MS, UV, IR	Inhibition of *β*-glucuronidase enzyme & cytotoxic	76
		14*α*-Hydroxy-etonogestrel			
		10*β*-Hydroxy-etonogestrel			
		11,22-Epoxy-etonogestrel			
		6*β*-Hydroxy-etonogestrel			
*Cunninghamella* sp. (Salicorn 5)	—	Ergosterol, stigmasta-7,22-diene-3*β*,5*α*,6*α*-triol and stigmasterol	Electrospray ionization mass spectrometry (ESI-MS)	ND^a^	63
*C. echinulata*		3-*O*-Formyl-27-hydroxyfusidic acid	1D, 2D-NMR, HRESIMS	ND^a^	70
*C. echinulata* NRRL 1382	Fusidic acid	Ergosterol	1D, 2D-NMR	ND^a^	69
*C. elegans*	Adrenosterone	11-Ketotestosterone	Single-crystal X-ray diffraction	ND^a^	77
*C. elegans*	Cortexolone	Prednisolone	NA^b^	ND^a^	78 and 79
		11*α*-Hydroxyprogesterone			
		Cortisol			

a ND: not determined.

b NA: not available.

### 3.5. Polysaccharides, polyphenols and quinones

**Fig. 9:**
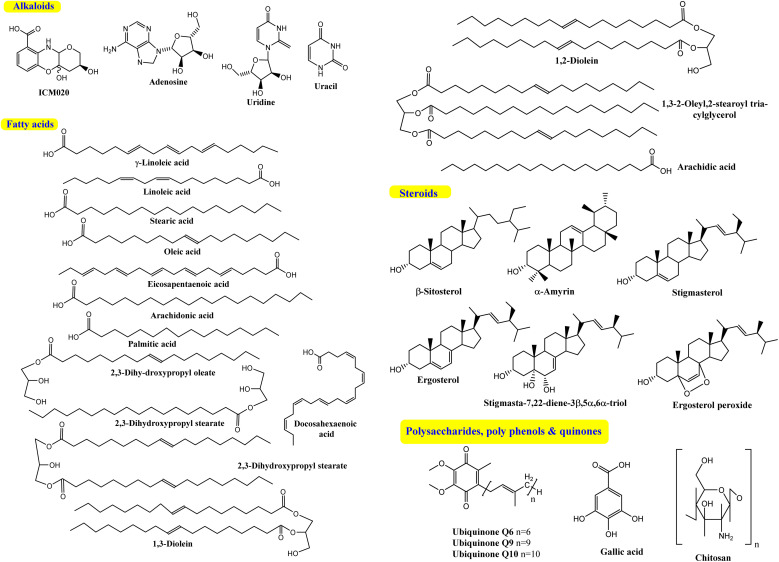
Secondary metabolites isolated from the *Cunninghamella* species.

*Cunninghamella* is thought to be a possible source for polysaccharides (Fig. 9) like chitosan, which can be recovered in significant amounts from fungal cell walls.^80^ Chitosan is also produced from other fungal sources, like *Rhizopus arrhizus*, *Mucor rouxii*, *Aspergillus niger*, *Penicillium notatum* and *Absidia orchidis*.^81^ Fungal chitosan is preferred over animal chitosan because the latter builds up in the renal tissue and increases the excretion of calcium in urine, which might result in kidney stone formation.^82,83^ Using three *in vitro* tests, fungal oligochitosan isolated from *C. elegans* was shown to exhibit more potent antioxidant activity than animal chitosan (reducing power, hydroxyl radical chelation and iron chelation) with a total antioxidant capacity of 17%, 40% and 13%, respectively. However, animal chitosan showed no activity in these tests.^84^ Gallic acid (GA) was also isolated from *C. elegans*, which induced a variety of biological activity to be described later.^51^

Another *Cunninghamella* metabolite, ubiquinone, was used as a biochemical marker for the classification and identification of *Cunninghamella* sp.^85^ This methodology helps with morphological taxonomy to tackle a number of issues pertaining to classification and phylogenetics. *Cunninghamella* has three types of ubiquinones based on the carbon numbers in their side chain (ubiquinone Q6, Q9 and Q10), as presented in Fig. 9. Ubiquinone Q6 was found in *C. bertholletie*, *C. elegans* and *C. ramosa*, while ubiquinone Q9 was found in *C. elegans*, *C. blakesleeana* and *C. echinulata*. However, *C. bertholletie* and *C. elegans* are the only producers of ubiquinone Q10.^86^ On the other hand, it is worth noting that the bio-transformed compounds are still missing, and further investigations are required.

## 4. Biological, agricultural, environmental, and industrial applications

**Fig. 10:**
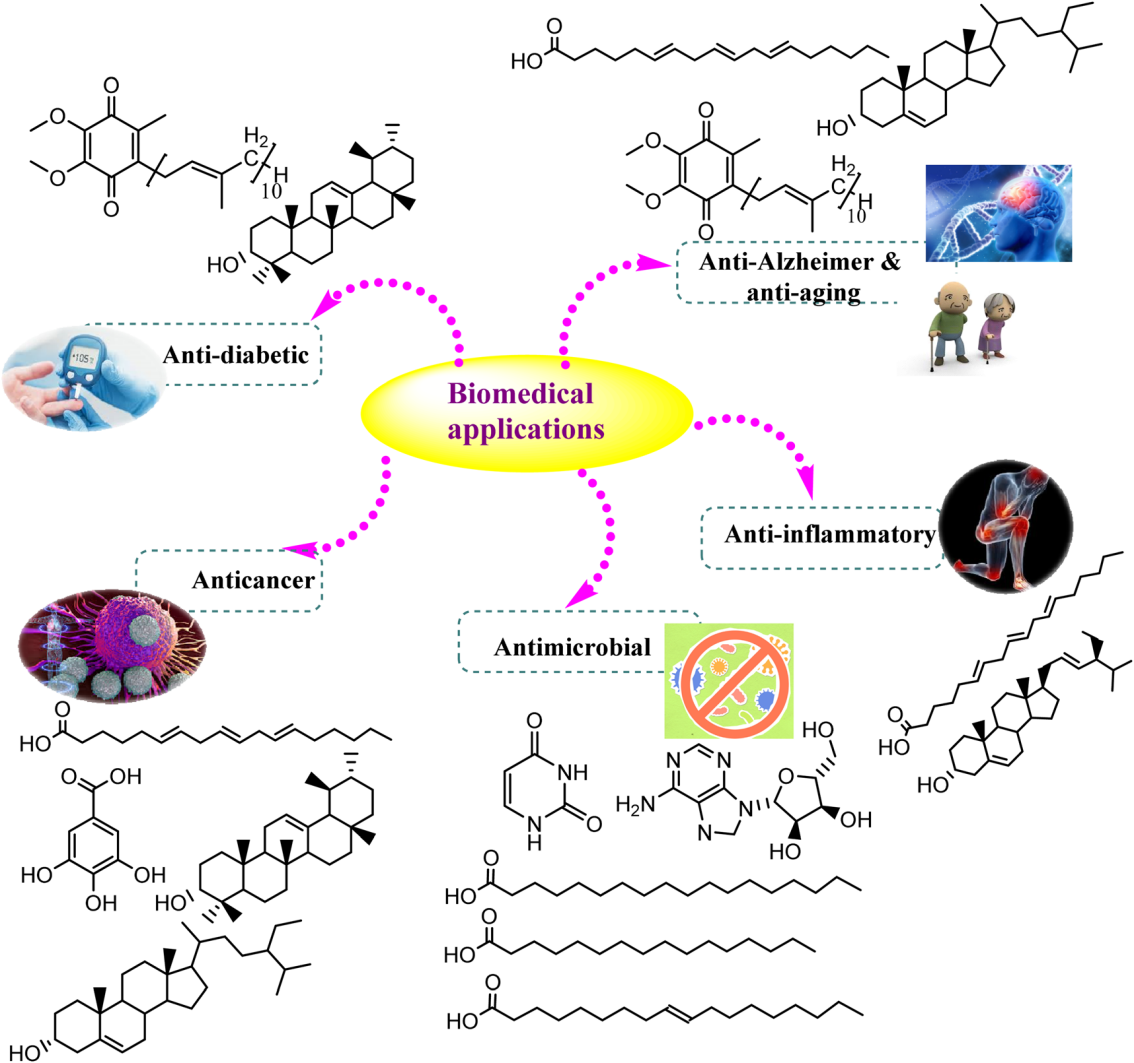
Biomedical uses of the chemically identified compounds derived from *Cunninghamella* species.

The secondary metabolites fabricated by endophytic fungi are well defined by their vital role in several applications. As the global population increases, the request for pharmaceutical and agricultural substances has dramatically increased. Hence, *Cunninghamella* sp. will gain tremendous attention in the future, particularly for the isolation of bioactive compounds.^87^ The current section will discuss the various biological, industrial, agricultural, and environmental values of previously isolated metabolites, and provide new insights into the recent applications of *Cunninghamella* sp. and as summarized in Table 4 and Fig. 10.

Summary of various biological activities for different isolated metabolites from *Cunninghamella* sp.

**Table:** 

Antimicrobial activity								
Isolated compounds	*Cunninghamella* sp.	Microorganism	Inhibition zone (mm)	MIC (μg mL^−1^	Standard	Inhibition zone (mm)	MIC (μg mL^−1^)	Ref.
Oleic acid	*C. blakesleeana*	*Salmonella typhimurium*	6.5 ± 0	700	Gentamycin	22.6 ± 1.5	01.95	65
		*Staphylococcus aureus*	13.0 ± 0.1	250	Ampicillin	22.00 ± 1.0	01.95	
	*C. elegans*		11.0 ± 0.3	—	Penicillin G	29.5 ± 0.8	—	91
Stearic acid	*C. blakesleeana*	*Salmonella typhimurium*	5.9 ± 0.9	750	Gentamycin	22.6 ± 1.5	01.95	65
		*Staphylococcus aureus*	11.0 ± 0.3	360	Ampicillin	22.00 ± 1.00	01.95	
	*C. elegans*		15.0 ± 0.5	—	Streptomycin	25.0 ± 0.2	—	91
Palmitic acid	*C. blakesleeana*	*Salmonella typhimurium*	8.0 ± 0.8	690	Gentamycin	22.6 ± 1.5	01.95	65
		*Staphylococcus aureus*	15.0 ± 0.5	200	Ampicillin	22.00 ± 1.00	01.95	
	*C. elegans*		13.0 ± 0.1	—	Penicillin G	29.5 ± 0.8	—	91
Adenosine	*C. elegans*	*Staphylococcus aureus*	30.0 ± 0.1	20	Penicillin G	29.5 ± 0.8	—	91
					Vancomycin	—	0.75	
Uridine	*C. elegans*	*Staphylococcus aureus*	11.0 ± 0.1	150	Penicillin G	29.5 ± 0.8	—	91
					Vancomycin	—	0.75	
Gallic acid	*C. elegans*	*Staphylococcus aureus*	5.0 ± 0.5	130	Penicillin G	29.5 ± 0.8	—	91
					Gentamicin	—	0.35	
Uracil	*C. elegans*	*Staphylococcus aureus*	7.0 ± 0.5	210	Penicillin G	29.5 ± 0.8	—	91
Glucose fatty acid esters	*C. echinulata*	*Bacillus subtilis*	14.1 ± 0.5	—	Eicosapentaenoic acid	17.0 ± 0.5	—	112
		*Candida albicans*	14.3 ± 0.0	—		20.0 ± 0.1	—	
		*Staphylococcus aureus*	14.1 ± 0.5	—		17.0 ± 0.2	—	
Chitosan	*C. elegans*	*Escherichia coli*	33.8 ± 1	0.375	—	—	—	90
		*S. aureus*	33.2 ± 1.3	0.375		—	—	
		*C. albicans*	23.5 ± 0.8	1.25		—	—	
		*Penicillium expansum*	8.2 ± 0.3	2.75		—	—

**Table:** 

Anticancer activity				
Isolated compounds	*Cunninghamella* sp.	Cancer cell line	IC_50_ (μM)	Ref.
GLA	*C. echinulata* and *C. blakesleeana*	HT-29 human colorectal cancer cell line	255	113
GA	*C. elegans*	SW480 and SW620 colorectal cancer cell lines	22.39, 11.8	98
*β*-Sitosterol	*C. elegans* and *C. blakesleeana*	HCT-116 colon cancer cell	140	99

**Table:** 

Anti-inflammatory					
Isolated compounds	*Cunninghamella* sp.	Cell line/animals	Dose	Inflammatory mediator affected	Ref.
Stigmasterol	Salicorn 5 (*Cunninghamella* sp.)	BEAS-2B human lung epithelial cell line	20 g mL^−1^	IL-13	101
Chitosan	*C. elegans*	Colonic homogenates of colitis mice	30 mg kg^−1^	TNF-α, IL6 and NF-kβ	104

**Table:** 

Anti-Alzheimer and anti-aging					
Isolated compounds	*Cunninghamella* sp.	Experiment	Dose/IC_50_	Enzymes/indicators affected	Ref.
GLA	*C. echinulata*	*In silico*	7.6 × 10^−5^ M	Amyloid cleaving enzyme (BACE1)	105
	*C. blakesleeana*				
Ubiquinone Q_10_	*C. bertholletie*	*In vitro*	15 μg mL^−1^	Senescence-associated secretory phenotype (SASP) indicators (p21, IL-8, CXCL1, and MMP-1)	106
	*C. elegans*

**Table:** 

Antidiabetic, antiplatelets and anti-hypercholesteremia					
Isolated compounds	*Cunninghamella* sp.	Experiment	Dose/IC_50_	Biological effect	Ref.
*α*-Amyrin	*C. elegans*	*In vivo*	5 and 10 mg kg^−1^	Antidiabetic	107
	*C. blakesleeana*			Anti-hypercholesteremia	
Ubiquinone Q_10_	*C. bertholletie*	*In vivo*	5 g kg^−1^	Antidiabetic	108
	*C. elegans*			Antioxidant	
GA	*C. elegans*	*In vitro* and *in silico*	9.07 μmol L^−1^	Antiplatelets	109
GLA	*C. echinulata*	*In vivo*	2.88 and 7.68 g kg^−1^	Anti-hypercholesteremia	110
	*C. blakesleeana*				
*β*-Sitosterol	*C. elegans*	*In vitro*	16 μM	Anti-hypercholesterolemia	111
				Antidiabetic	
	*C. blakesleeana*			Antioxidant	

### 4.1. Biological activities

Fungal secondary metabolites are of intense interest due to their considerable biological activities.^25–27^ A strong relationship has been created between the pharmaceutical and biochemical industries with endophytic fungi, in particular *Cunninghamella*, due to the abundance of several promising metabolites that have various biological values, like anti-cancer, antioxidants, antidiabetic, anti-inflammatory and antimicrobial values (Table 4). These values offer significant potential for their usage in medicine.^88^

#### 4.1.1. Antimicrobial activity

*Cunninghamella* sp. has become a focal point of interest as one of the principal sources for the biodiversity of secondary metabolites used as antimicrobial agents. A number of relevant studies have investigated the antimicrobial activity of the isolated compounds with an emphasis on fatty acids. For instance, GLA stands out among the fatty acids due to its wide range of biological activities, which include its ability to fight off a variety of Gram-negative periodontal pathogens, particularly *Prevotella intermedia* and *Porphyromonas gingivalis*, when triclosan (a broad-spectrum antimicrobial agent found in toothpaste) is used as a positive control. The study revealed that GLA was more potent against the tested pathogens with a minimum inhibition concentration (MIC) of 39.06 and 9.76 μg mL^−1^, respectively, compared to triclosan (1250 and 78.12 μg mL^−1^, respectively) against the same pathogens.^89^ GLA was also shown to be active against several oral pathogens like *Aggregatibacter actinomycetemcomitans*, *Candida albicans*, *Fusobacterium nucleatum*, and *Streptococcus mutans*.^89^ In addition, another study has reported on the antimicrobial activity of isolated fatty acids against *Staphylococcus aureus*, *Pencillium expansum* and *Salmonella typhimurium*. The results showed that stearic acid was the most potent compound against *S. aureus* with a zone of inhibition of 15 ± 0.5 mm at MIC of 360 μg mL^−1^, compared to palmitic acid and oleic acid (13.0 ± 0.1 and 11.0 ± 0.3 mm, respectively). Palmitic acid and oleic acid showed significant antimicrobial efficacy against *S. typhimurium* and *P. expansum* with a zone of inhibition of 8 ± 0.8 and 8.5 ± 0.6 mm at MIC of 690 and 660 μg mL^−1^, respectively, compared with stearic acid with the use of gentamycin, ampicillin, and amphotericin B as standard antimicrobial drugs, as introduced in Table 4.^65^ Furthermore, chitosan isolated from *C. elegans* showed antimicrobial activity against *Escherichia coli*, *S. aureus* and *C. albicans* with zones of inhibition of 33.8, 38.2 and 23.5 mm at MIC of 0.57, 0.37 and 1.25 mg mL^−1^, respectively.^90^ Oral administration of an ethanol extract of *Cunninghamella* sp. (400 mg kg^−1^) resulted in potential safety for up to 5000 mg kg^−1^, and no problems appeared in the kidney or liver functions.^65^ In the same context, an alcoholic extract of *C. elegans* induced activation against Gram-positive and Gram-negative bacteria,^91^ while an ethyl acetate extract showed the highest efficacy against *S. aureus*, followed by an ether extract with an inhibition zone at 29.2 ± 0.08 and 26.2 ± 0.08 mm, respectively. The lowest activity was obtained by a butanol extract (2.4 ± 0.1 mm), followed by an ethyl acetate extract (3.4 ± 0.5) against *Streptococcus pyogenes*.

Adenosine was evaluated for its antimicrobial activity, and the results indicated that adenosine was most active against *S. aureus* with an inhibition zone of 30 ± 0.1 mm at a concentration of 20 μg mL^−1^ compared to those of streptomycin and penicillin G as standard antibiotics (25 ± 0.2 and 29.5 ± 0.8 mm, respectively).^51^ Additionally, the topical application of 1 mg mL^−1^ adenosine to an experimentally excised wound surface sped up the healing process, according to a study on the wound-healing properties of adenosine isolated from *C. elegans*. Topical application of adenosine showed 76.5% wound correction after 14 days since treatment started, which was close to that of the standard drug latmoxef (100%). Complete wound correction by adenosine occurred after 18 days.^51^

Moreover, glucose esters of different fatty acids from C. *echinulata* were synthesized using lipases as biocatalysts. The biological activity assay of glucose fatty acid esters from *C. echinulata* indicated that they were efficient against *Bacillus subtilis*, *Candida albicans*, and *Staphylococcus aureus* at MIC 40 μg mL^−1^ with inhibition zones of 14.1, 14.3 and 12.3 mm, respectively. The results revealed the high pathogenic activity of fatty acid esters present in *Cunninghamella* sp. compared to those founded in *U. isabelline*. This could be attributed to GLA present in the lipids of *C. echinulata*, which has been recognized for its antibacterial activity in higher amounts. Furthermore, the glucose fatty acid esters from *C. echinulata* demonstrated notable insecticidal action against *Aedes aegypti* larvae with LC_50_ of 0.54 mg L^−1^. Additionally, after treatment with 10 g mL^−1^ of glucose esters, the SKOV-3 ovarian cancer cell line experienced a high proportion of apoptosis (39.2%).^92^ Furthermore, *β*-sitosterol exhibits potent antiviral activity against the influenza A virus. A study by Shokry *et al.* showed that *β*-sitosterol demonstrated promising antiviral efficacy against A/H1N1 and A/H5N1 strains with IC_50_ of 0.975 and 0.295 μg mL^−1^, respectively, compared to zanamivir as a positive control. It has been discovered that β-sitosterol can influence various viral replication processes, including viral adsorption and replication. The significant inhibitory impact of *β*-sitosterol against the hemagglutinin surface protein and neuraminidase, with docking energies of −6.40 and −29.40 kcal mol^−1^, was attributed as the mechanism of *β*-sitosterol's antiviral activity.^93^

The antiparasitic efficacy of α-amyrin was *in vitro* testing against *Trypanosoma cruzi*. The study showed that the isolated compound was more effective against the amastigote stage than the trypomastigote stage with an IC_50_ value of 9.08 μg mL^−1^ compared to that of the reference medication nifurtimox (3.07 μg mL^−1^). A molecular docking study indicated that α-amyrin has a greater affinity to *T. cruzi* cysteine synthase (TcCS) with a binding energy of −9.8 kcal mol^−1^.^94^

In conclusion, from the tabulated data (Table 4), it was clearly observed that most of the antimicrobial research involved very few species of *Cunninghamella*, *e.g.*, *C. elegans*, *C. echinulata* and *C. blakesleeana*. In addition, fatty acids exhibited the highest antimicrobial activity compared to other classes. This is consistent with previous literature data, which confirmed the potential antimicrobial activity of fatty acids. However, further investigations are recommended with the incorporation of new active species of *Cunninghamella*, aiming to isolate new lead compounds.

#### 4.1.2. Anticancer

GLA has demonstrated anticancer activity in patients with breast cancer. Due to its capacity to lower estrogen receptor expression and enhance the inhibitory effects of tamoxifen through enhanced downregulation of estrogen receptor-mediated growth, it has been demonstrated that GLA (at a dose of 2.8 g per day) can be used as an adjunct to primary tamoxifen (at a dose of 20 mg per day) in cases of endocrine-sensitive breast cancer.^95^ Occasionally, some of the patients would not have the ability for natural GLA production, and this would lead to using GLA dietary supplements. The high productivity of GLA was enhanced in *C. echinulata* that had been treated with a pulsed strong magnetic field *via* physical mutagenesis technology. Yields of GLA (Mut-29, Mut-15, Mut-64) were improved by 46.2%, 23.1%, and 19.2%, respectively, compared to the parent strain.^60^ On the other hand, GLA was successfully incorporated in fatty acid lithium salt to increase its solubility. Consequently, it could be smoothly administrated to the cell.^96^ It was found that using lithium gamma-linolenate enhanced the permeability of cancer agents and improved the cytotoxic activity of GLA.^97^ Two dosage levels were used (either 0.84 g kg^−1^ (*n* = 1) or 0.28 g kg^−1^ (*n* = 2)) with the oral treatment of one dose value of a total of 10.5 g per patient (*n* = 2). When compared with conventional regimens, treatment with GLA indicated the survival of pancreatic cancer patients that showed a potential result.^97^

To maximize the impact of the anticancer efficacy in *Cunninghamella* sp., a phenolic acid such as gallic acid (GA) was previously isolated from *C. elegans*.^51^ Gallic acid has a wide variety of biological activities. Recently, GA has shown strong cytotoxic effects on colon cancer cell lines. Three different types of cell lines, representing various stages of cancer severity, were used to assess GA's cytotoxic effect: colon epithelial cells CRL1790, which represent the non-tumorigenic stage; and colorectal cancer cell lines SW480 and SW620, which represent the primary tumor stage and the aggressive metastatic stage, respectively. The results revealed that GA inhibited the cell growth of SW480 and SW 620 at IC_50_ of 22.39 ± 2.12 and 11.8 ± 1.5 μM, respectively. Furthermore, GA showed high selectivity toward cancer cells rather than non-cancer cells due to the high IC_50_ (>100 μM) of GA against CRL1790 cells. Moreover, the results of the cell cycle analysis of the tested cell lines showed that GA induced prominent S and G_2_/M phases. GA changed the frequency of the cells from 34.2% to 43.8% at the S phase, and 7.7% to 14.5% at the G_2_/M phase. These results indicate that GA may affect DNA replication, inducing cell cycle arrest in the S and G2/M stages. These findings were supported by an *in vivo* experimental model, and immunofluorescent analysis of the tumor tissues taken from sacrificed mice showed that GA had a downregulating effect on several G-Quadruplexes (G4)-enriched oncogenes, leading to DNA damage.^98^

Additionally, *β*-sitosterol significantly inhibits colon cancer cell growth, which has the ability to suppress HCT-116 cell proliferation. The mechanism was studied, and it was found that β-sitosterol downregulates the gene and protein expression of lymphoid enhancer binding factor (LEF1), which is an oncogenic gene. In addition, it disrupts Wnt/β-catenin pathway transmission in HCT-116 at the same concentration.^99^ These findings indicated that *Cunninghamella* may be a valuable source for potential anticancer agents (Table 4).

#### 4.1.3. Anti-inflammatory

Stigmasterol was isolated from *Cunninghamella* sp. (Salicorn 5), which is an endophytic oleaginous fungus from *Salicornia bigelovii* Torr.^100^ This compound was isolated and identified by means of ESIMS and NMR techniques. One of stigmasterol's more recent biological functions is its ability to reduce inflammation in asthma by inhibiting neurokinin-1 receptors (NK1-R), which are found in the smooth muscles of the airways and submucosal glands, and are crucial for the production of mucus and the tension of the muscles. Using immunofluorescent labelling and western blot techniques, Zhang *et al.* discovered that stigmasterol at a concentration of 20 g mL^−1^ displayed an anti-inflammatory effect by reducing NK1-R expression in interleukin 13 (IL-13) induced human lung epithelial BEAS-2B cells.^101^ Another report investigated the anti-inflammatory effect of GLA against rheumatoid arthritis (RA). Treating RA patients for 24 weeks with a total daily dose of 1.4 g of GLA led to a significant decrease in the disease's signs and symptoms.^102^ This may be attributed to the presence of GLA, which quickly transforms into di homo gamma linoleic acid, a precursor of prostaglandin E, which is an eicosanoid with anti-inflammatory and immunoregulatory properties (Table 4).^103^

On the other hand, several applications of chitosan were examined for the treatment of various ailments. In experimental colitis, chitosan was discovered to have anti-inflammatory properties. Using ELISA kits, Jhuundoo *et al.* discovered that 30 mg kg^−1^ of chitosan significantly decreased the levels of myeloperoxidase, alkaline phosphatase, TNF-*α*, IL6 and NF-k*β* in the colonic homogenates of colitis mice compared to untreated mice.^104^

#### 4.1.4. Anti-Alzheimer and anti-aging

The anti-Alzheimer activity of GLA was evaluated utilizing the *in silico* docking approach. The results indicated that GLA exerted a considerable and targeted inhibitory impact against the B-side of the amyloid cleaving enzyme (BACE1) with an IC_50_ value of 7.6 × 10^−5^ M, and strongly interacted with the allosteric site of the enzyme at the OH group of CYS359 ^107^.

Recently, the impact of ubiquinone Q10 was potentially investigated for anti-ageing activity, and showed significant results. Ubiquinone Q_10_ biosynthesis decreases with age in different tissues, including the skin. However, it could be modulated by 3-hydroxy-3-methyl-glutaryl-coenzyme A (HMG-CoA) reductase inhibitors such as statins, which resulted in a senescence phenotype. Ubiquinone Q10's impact in the process of skin ageing was studied by Marcheggiani *et al.* using statin-pretreated cultured human dermal fibroblasts (HDF). The outcomes showed that statin-treated HDF could be prevented from developing senescence and ageing indicators, and could even be saved by ubiquinone Q10 supplementation at a dose of 15 μg mL^−1^. Along with increasing the extracellular matrix's components including elastin and collagen type 1, it greatly decreased several senescence-associated secretory phenotype (SASP) indicators like p21, IL-8, CXCL1, and MMP-1 (Table 4).^106^

#### 4.1.5. Anti-diabetic, anti-platelet and anti-hypercholesteremia

*α*-Amyrin was investigated as a potential anti-diabetic medication. According to the study, when given to streptozotocin-induced diabetic rats, *α*-amyrin at doses of 5 and 10 mg kg^−1^ could lower plasma glucose, total cholesterol, triglycerides, and low-density lipoprotein (LDL), while increasing high-density lipoprotein (HDL) levels. Additionally, it could lower liver enzyme levels (SGOT and SGPT) to levels that are nearly normal.^107^

Another investigation also demonstrated that ubiquinone Q10 has strong anti-inflammatory, antioxidant, and anti-diabetic properties in streptozotocin-induced diabetic rats. The findings showed that diabetic rats treated for 21 days with 5 g kg^−1^ in rat food experienced a significant decrease in blood glucose, IL-6, malondialdehyde (MDA), and myoglobin levels. These findings indicated that ubiquinone Q10 may have a beneficial effect on diabetes complications.^108^

In addition, the antiplatelet aggregation activity of GA was verified *via* different strategies, including surface plasmon resonance (SPR), molecular docking, molecular dynamics simulation with a thrombin inhibition assay. According to the findings, GA can inhibit thrombin with an IC_50_ of 9.07 μmol L^−1^, which in turn reduces thrombin-induced platelet aggregation by 35% when compared to cells that have been treated with thrombin. This result was brought on by the thrombin-GA equilibrium system's high binding free energy (−14.6 kcal mol^−1^).^109^

Moreover, the anti-hypercholesteremic action of GLA was investigated. It was proved that GLA can lower the body fat content by inducing the activities of liver carnitine palmitoyl transferase and peroxisomal *β*-oxidation for fatty acids in the liver.^110^ Sterols from natural sources have various biological activities.^51,100^ Vasanth *et al.* recently investigated the anti-adipogenicity of *β*-sitosterol. It was found that *β*-sitosterol reduced the viability of 3T3-L1 mouse fibroblast cells in a dose-dependent way by suppressing the cell cycle stages (particularly S and G_2_/M) with a considerable reduction in the intracellular lipid accumulation and an increase in the glucose uptake. Additionally, the findings demonstrated that *β*-sitosterol decreased the formation of reactive oxygen species from 91.65% to 42.97% at a concentration of 16 μM (Table 4).^111^

### 4.2. *Cunninghamella* metabolites of agricultural, environmental, and industrial values

Recently, the industrial and environmental applications of naturally occurring compounds have gained tremendous interest to produce green and sustainable substances. Chitosan is one of the most crucial compounds to be widely applied in different sectors, including food and beverages, wastewater treatment, biopharmaceutics, cosmetics and toiletries, and agricultural uses.^114^ The biological activity of chitosan is significantly dependent on its molecular weight and molecular weight distribution (100 < *M*_w_ < 500 kDa).^115^

As a bio-protector and substitute for traditional fertilizers, chitosan from the *Cunninghamella* fungi can be used. Chitosan's efficiency against tomato wilt caused by *Ralstonia solanacearum* bacterium was compared to conventional fertilizers in a study. According to the findings, plants exposed to conventional fertilizers (NPKF) began to exhibit severe disease symptoms one week after being infected with *R. solanacearum*, and all the plants perished two weeks later. However, plants treated with chitosan developed improved plant traits and bacterial disease resistance.^116^ Moreover, chitosan isolated from *C. elegans* revealed a suitable inhibition activity of 81.7% against mycelial growth of *Scytalidium lignicola*, which causes potential decrease in cassava production all over the world.^117^

In another study, chitosan from *C. elegans* was found to have potent fungicidal activity against *Fusarium oxysporum* f. sp. tracheiphilum, which is a pathogenic fungus responsible for one of the most frequent diseases in cowpea (*Vigna unguiculata* L.) crops. This has great socioeconomic importance, especially in Brazil, because it represents a popular dietary source of protein, carbohydrates, and iron, and can be used for animal feed and for the recovery of soil fertility as green manure. This pathogen causes a disease known as Fusarium wilt, which can lead to a reduction in plant growth, chlorosis, wilting and premature leaf fall, all of which almost inevitably lead to the death of the afflicted plants. The results indicated that higher concentrations of fungal chitosan (4.0–6.0 mg mL^−1^) were responsible for the lowest Fusarium wilt disease severity index in cowpea plants. This was because these higher chitosan concentrations directly induced catalase and peroxidase activity in plants, which in turn controlled the reactive oxygen species equilibrium for plant resistance, and led to a significant decrease in the disease severity in cowpea. These results are crucial for establishing sustainable agriculture and avoiding the usage of pesticides.^118^

Additionally, fungal chitosan has an inhibitory effect against *Botrytis cinerea* and *Penicillium expansum*, which can deteriorate the fruit crop of table grape after harvesting. Chitosan can inhibit the mycelial growth of *B. cinerea* and *P. expansum* at MIC of 15 mg mL^−1^ at 80.4% and 85.7%, respectively. Moreover, chitosan can inhibit spore germination of the two previous fungi at 98.2% and 94.3%, respectively, at the same MIC.^119^ Consequently, chitosan could be used effectively for extending the shelf life of foods and keeping them safe for a long time. As a bio-preservative in processed fish sausages made from Nile tilapia (*Oreochromic niloticus*), fungal chitosan also plays a positive role in the food sector. The results showed that fish sausages treated with 1.5% chitosan significantly reduced the microbiological load of coliforms, yeasts, molds, *E. coli*, and *S. aureus*, while maintaining the sensory quality of the sausages for a 28 days storage period at 4 °C. Mean ratings for the odor, taste, color, and texture of the chitosan-treated samples from the panelists were 93.2%, 88.3%, 92.1%, and 89.8%, respectively. The control samples' comparable scores, on the other hand, had mean values of 71.4%, 64.2%, 87.9%, and 81.1%, respectively.^120^

**Fig. 11:**
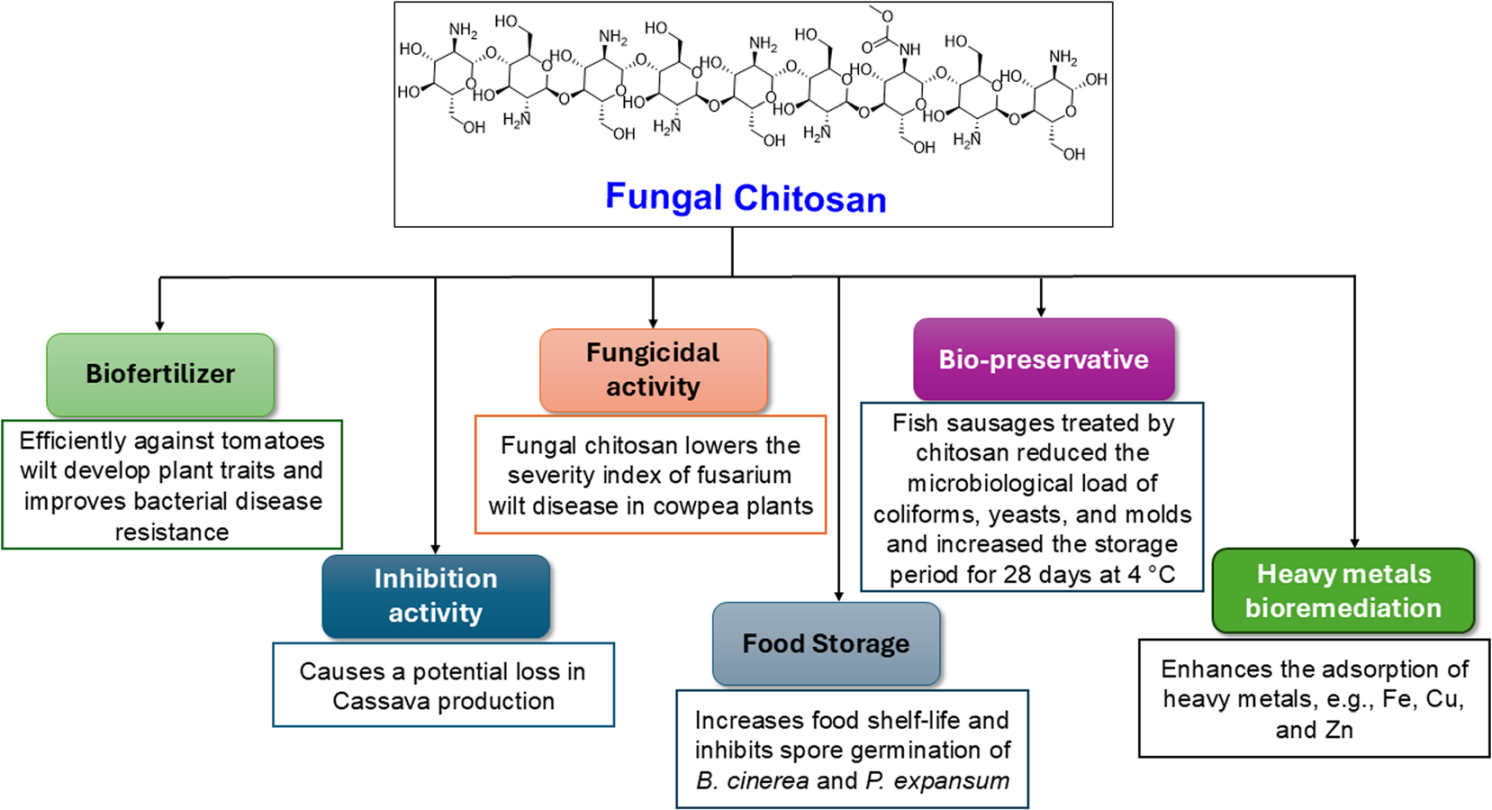
Agricultural, environmental, and industrial values of chitosan isolated from *Cunninghamella* species.

The environmental value of fungal chitosan and chitin was represented by the highest affinity of chitosan and chitin from *C. elegans* for copper and iron adsorption, respectively.^121^ Chitin is the parent compound of chitosan that is found in several organisms, forming the exoskeletons of crustaceans, mollusks, insects, algae and the cell wall of fungi.^122,123^ Additionally, chitosan was demonstrated to be a powerful metal adsorbent for zinc and lead ions, and its adsorption capacity significantly increased as the metal concentration increased.^90^ This affinity made them potential agents for heavy metal bioremediation in polluted environments, as shown in Fig. 11.

## 5. Different enzymes from *Cunninghamella* species

The swift advancements of biotechnology and discoveries in the field of enzymology have triggered great curiosity toward enzyme preparations. The *Cunninghamella* genus possesses diverse biosynthetic machinery, which include crucial genes and enzymes utilized in the biotransformation and modification of different organic compounds.^124^ The enzymatic system in *Cunninghamella* can be divided into two classes based on their responsibilities and relation with mammalian systems.^125^ It includes enzymes responsible for biotransformation or xenobiotic metabolism, while the second category includes enzymes with different functions and is incorporated in industrial usage.^125^ Currently, enzymes derived from *Cunninghamella* sp. are involved in a variety of applications encompassing industry, agriculture, medicine, and bioremediation.

### 5.1. *Cunninghamella* enzymes of biological values

**Fig. 12:**
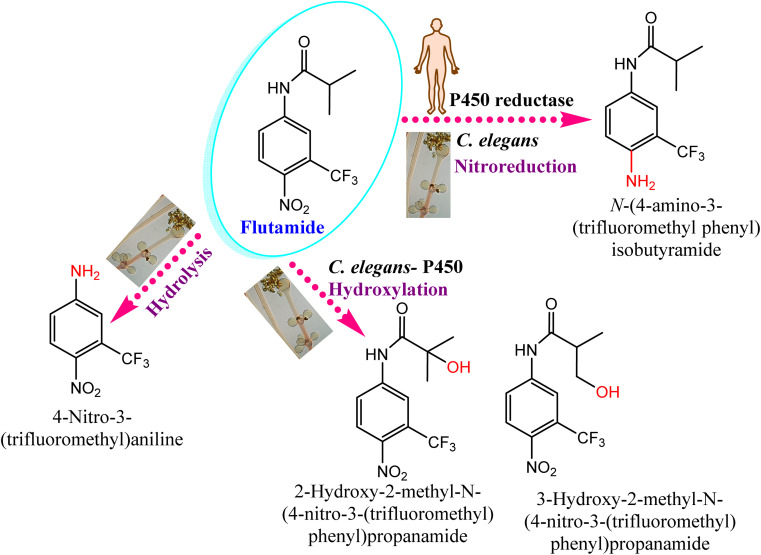
Effect of the CYP-450 reductase enzyme on flutamide biotransformation. Flutamide biotransformed by *Cunninghamella elegans* produces the same metabolites as those present in humans.

*Cunninghamella* sp. have several enzymes comparable to the human enzyme system such as CYP-450 enzymes, which can metabolize a variety of medicines and transform them into biologically active forms.^126,127^ In that context, *Cunninghamella* has the capacity to catabolize xenobiotic chemicals into products that can then be derivatized by additional chemical reactions to create metabolites with unique biological functions. The gene encoding for CYP 5313D1 is thought to play a role in the metabolism of xenobiotics such as the non-steroidal anti-inflammatory medicine flurbiprofen, according to *in silico* analysis and expression studies. The CYP 5313D1 gene was obtained from *C. elegans*. When it was heterologously overexpressed in yeast (*Pichia pastoris*), a recombinant strain that metabolized flurbiprofen to 4′ hydroxy flurbiprofen was identified using GC-MS analysis. This metabolite is the same one produced by *C. elegans* when flurbiprofen is used as a substrate.^26^ It is possible to derivatize this hydroxylated metabolite utilizing the Mannich base reaction to create a variety of compounds that work as multifunctional drugs with strong anti-platelet aggregation, anti-neuroinflammatory, and antioxidant properties.^128^ In addition, a recent study investigated the impact of CYP-450 reductase enzyme derived from *C. elegans* against the nitro-reduction of flutamide.^129^ The results induced the potential conversion of flutamide as an anticancer drug to the nitro-reduced metabolite, which is similar to that produced from the same substrate in human NADPH: CYP-450 reductase shown in Fig. 12.^129^ The nitro reductase effect was prolonged to other substances like nilutamide and environmental pollutants, *e.g.*, 1,3-dinitronaphthalene and 1-nitronaphthalene. Comparative studies with cell lysates of recombinant yeast showed the high sensitivity of reductases toward oxygen.

**Fig. 13:**
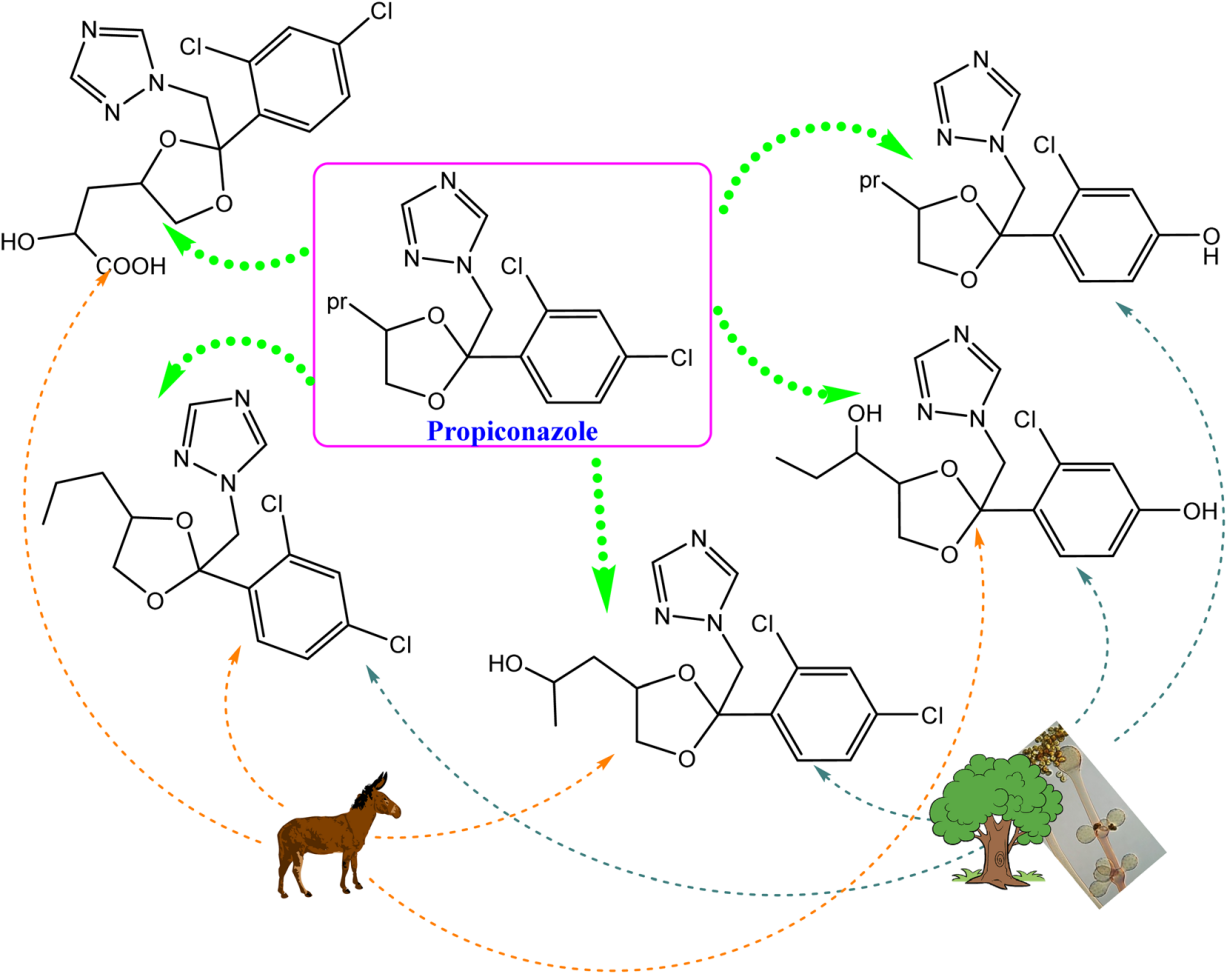
Comparison of the propiconazole breakdown and metabolic pathways between *Cunninghamella elegans* and other environmental sources, *e.g.*, soil, plants, and animals.

Following the action of the CYP-450 enzyme in the same species, the metabolic breakdown of propiconazole was recently investigated.^130^ The enzyme initially performs hydroxylation and oxidation reactions of propyl groups in phase I metabolism. Five metabolites were accumulated after 3 days of post-treatment, and indicated that 98% of propiconazole was approximately degraded.^130^ Interestingly, the formed metabolites were comparable to previously identified compounds from other natural sources, *e.g.*, animals, plants and soil, as presented in Fig. 13.

Another study involved *C. blakesleeana* for improving the low oral bioavailability of paeoniflorin.^131^ Paeoniflorin is a glycoside compound isolated from *Paeonia lactiflora*, and has various pharmacological effects, including diabetes mellitus-associated macrovascular complications,^132^ neuroprotective, antidepressant,^133^ anti-inflammatory,^134^ anti-Parkinson,^135,136^ and anti-Alzheimer effects.^137,138^ Using a comparison of mass spectroscopy and transcriptomics, the gene (G6046) for a paeoniflorin-converting enzyme was extracted from *C. blakesleeana*. It was investigated whether optimizing the conditions would promote the highest enzyme activity. When the enzyme activity for paeoniflorin metabolism was tested *in vitro*, benzoic acid and other benzoate substances were produced, which may make them easier to absorb into the bloodstream, pass through the blood–brain barrier, and enter the central nervous system, where they can exert the pharmacological effects previously mentioned with the greatest ease.^139^

Moreover, *Cunninghamella* can produce the catalase enzyme, which is considered a potent antioxidant defense enzyme.^140^ Exogenous catalase generated from microorganisms like *Cunninghamella* can be added to the diet as an exogenous supplement to boost immunity against problems brought on by oxidative stress. Exogenous catalase from microorganisms was added to the meal in an *in vivo* experiment to see if it could reduce the damage that lipopolysaccharides (LPs) caused to the intestinal mucosa of weaned pigs. The findings demonstrated that feeding pigs a diet supplemented with 2000 mg kg^−1^ of exogenous catalase for 35 days helped to reduce the negative effects that LPs had on the intestinal mucosa. This was done by increasing the amount of catalase and super oxide dismutase in the intestines, and lowering the levels of malondialdehyde and H_2_O_2_ in the blood. The amounts of proinflammatory cytokines, such as tumor necrosis factor alpha (TNF-*α*) and interleukin 6 (IL-6), are also reduced by exogenous catalase supplementation of the food by 11.82% and 15%, respectively. Additionally, compared to pigs merely receiving LP treatment, exogenous catalase in the meal enhanced secretory immunoglobulin A content by 18.14%.^141^

### 5.2. *Cunninghamella* enzymes of agricultural, environmental, and industrial values

**Fig. 14:**
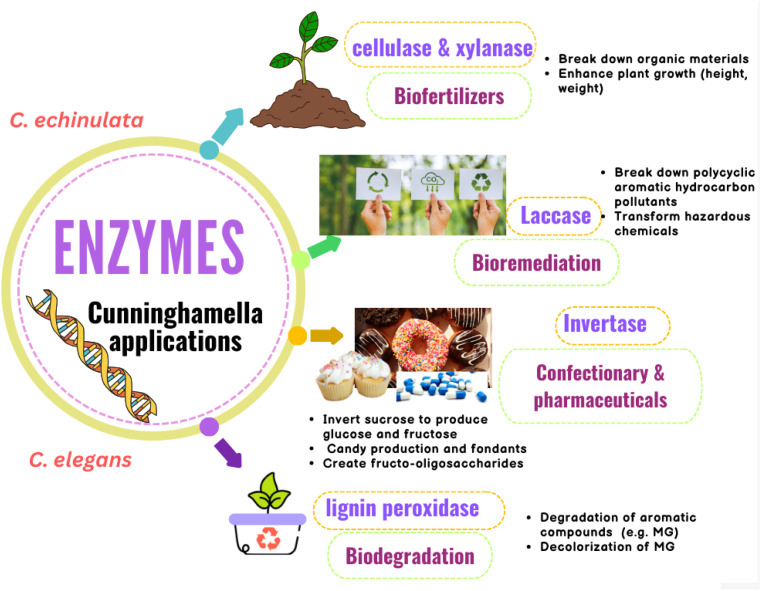
Diagram representing the agricultural, environmental and industrial applications of *Cunninghamella*-derived enzymes with their impacts.

Many *Cunninghamella* sp., such as *C. echinulata* and *C.* SL2 species, were proved to produce cellulase and xylanase enzymes using cellulose and xylan degradability *in vitro* methods.^142–144^ Thiep *et al.*^145^ studied the use of these enzymes from *Cunninghamella* SL2 sp. as biofertilizers to improve the soil for tea and Arabica coffee plants. According to the findings, *Cunninghamella* SL2 sp. exhibited significant enzymatic activity for cellulose and xylan with a clear zone of 22.5 and 24.4 mm, respectively. According to the study, these enzymes can break down organic materials and be employed as biofertilizers, as shown in Fig. 14. In comparison to the control plants (45.7 cm per plant, 5.6 g per plant, and 68.9 g per plant), they enhanced the plant height, root weight, and plant weight of coffee plants to 55.8 cm per plant, 9.2 g per plant, and 100.9 g per plant, respectively. Additionally, compared to the control (37.5 cm per plant, 2.3 g per plant, and 8.5 g per plant), these enzymes enhanced the plant height, root weight, and plant weight of tea plants to 54 cm per plant, 5.9 g per plant, and 22.3 g per plant, respectively. As a result, *Cunninghamella* sp.’ cellulase and xylanase enzymes can be utilized as biofertilizers to enhance the characteristics of the soil and the growth of plants.^145^

*Cunninghamella* produces laccase, a key lignin degradation enzyme that can break down polycyclic aromatic hydrocarbon pollutants (PAHs). Thus, it can be used in bioremediation strategies and has been found to be able to transform a wide range of hazardous chemicals, as tabulated in (Table 5).^146^ These hydrocarbons are continuously increasing due to industrial expansion, posing numerous risks to people, including the development of cancer and toxicity. According to a study to assess the laccase enzyme activity of *C. echinulata*, it has a strong ability to degrade three-ring PAHs (anthracene and phenanthrene) as well as phenolic compounds, with breakdown percentages ranging from 96.03% to 99.98% at various levels of PAHs (1%, 2%, 3%, 4% and 5%). High PAH concentrations were observed to boost the activity of the laccase enzyme. In addition, *Cunninghamella* might use PAHs as a source of carbon for growth.^147^

**Table:** Examples of the most important enzymes derived from the *Cunninghamella* species with applications

*Cunninghamella* sp.	Enzyme	Application	Ref.
*Cunninghamella* sp.	Cytochrome P450 (CYPs)	Antioxidant, metabolism of xenobiotics, anti-platelets aggregation, anti-neuroinflammatory	26 and 128
*C. elegans*	Cytochrome P450 reductase	Nitroreductase activity	129
*C. blakesleeana*	Catalase	Antioxidant	140
*C. echinulata* and *C. SL2*	Cellulase and xylanase	Biofertilizers	142–144
*C. echinulata*	Laccase	Bioremediation	146
*C. elegans*	Lignin peroxidase	Biodegradation of lignin-related aromatic compounds, *e.g.*, dye malachite green (MG)	149
*C. echinulata*	Invertase	Confectionary & pharmaceutical industry	157 and 158

Additionally, it was shown that the lignin peroxidase enzyme is essential for the biodegradation of lignin-related aromatic compounds, including the dye malachite green (MG), which is an *N*-methylated diamino triphenyl methane dye.^148^ It can be used to dye cotton, silk, wool, jute, leather, and pottery. MG is extremely harmful to mammals, especially humans.^149^*Cunninghamella* sp. are considered important sources for lignin peroxidase enzymes.^150^ Roushdy *et al.* studied the lignin peroxidase effect from *C. elegans* in the decolorization of MG. The findings demonstrated that in the presence of 5 mL of a *C. elegans* cell-free extract containing lignin peroxidase enzyme, 100% decolorization of MG was seen at doses of 10, 20, and 50 mg L^−1^ of MG. Additionally, the results showed that static conditions were better when compared to shaking conditions because in aerobic conditions, oxygen and dye competed for the decreased electron carriers.^151^

*Cunninghamella* sp. was found to have the ability of invertase enzyme production.^152^ Invertase is a hydrolyzing enzyme that is capable of breaking down the α-1,4 glycosidic linkage between d-glucose and d-fructose of sucrose.^153^ Due to the colored byproducts produced by acid hydrolysis processes, such as hydroxy methyl furfural, which is harmful to humans, enzyme hydrolysis of sucrose is preferable to acid hydrolysis.^154,155^ A study demonstrated that *C. echinulata* produces invertase enzyme at a high level.^152^ The conditions were ideal for optimum enzyme synthesis, and the fungus produced a lot of enzymes in culture media supplemented with apple peel. Due to its capacity to invert sucrose to produce a glucose and fructose mixture known as invert syrup, which is sweeter than sucrose due to the high sweetness of fructose, the invertase enzyme has a wide range of industrial applications. These applications comprise the confectionary industry and pharmaceutical industry, and include the formulation of drugs, digestive tablets, and cough syrups, as shown in Fig. 14.^156^ Due to its hygroscopic character, it can also be employed as a humectant in the production of candy and fondants. Invertase can also create fructo-oligosaccharides, which are great for diabetic patients because they have a lower calorie content while maintaining a similar sweetness.^157,158^

## 6. Conclusion

Endophytic fungi are increasingly recognized as a significant source of useful metabolites, which can be distinct derivatives that are either more potent or have identical potency to those of their host plants. Among the most well-known endophytes is *Cunninghamella*, which is attributed to its abundance of isolated metabolites and its capacity to biotransform a wide range of substrates into more active forms with a variety of biological effects, such as antimicrobial, anticancer, anti-inflammatory, anti-Alzheimer, antiaging, antidiabetic, antiplatelet, and antihypercholesteremia effects. Under the deep investigation for rediscovering the chemical profile of *Cunninghamella*, it was found to be superior in the biodiversity of FA production. However, none of the elucidated flavonoids have been reported yet, while a few alkaloids, steroids, polyphenols and polysaccharides were isolated. In contrast, *Cunninghamella* was distinguished in the biotransformation process. It revealed successful results in producing significant and rare metabolites, including steroids, alkaloids and flavonoids when compared to other classes like FAs, polyphenols and quinones, which have yet to be verified *via* biotransformation. These results inspired researchers to further study the chemistry of the *Cunninghamella* species, with the aim of answering these contradictions to discover new lead compounds. The current study rediscovered the potential applications of *Cunninghamella* sp. and associated metabolites in the context of different biological, agricultural, industrial and environmental disciplines. In addition to being a significant source of valuable compounds, *Cunninghamella* is also thought to be a valuable source of important enzymes, such as lignin peroxidase, catalase, cellulase, xylanase, laccase, and CYP 450, which have a variety of uses in industry, agriculture, medicine, and the environment. However, the use of *Cunninghamella* as a host candidate for heterologous expression of valuable proteins has not been attempted. Future work is recommended to focus on exploiting *Cunninghamella* sp. in more complicated biotechnology applications other than the perspective and retrospective studies used in drug discovery.

## Data availability

No data were used to create the information presented in the present research.

## Conflicts of interest

The authors declare no conflict of interest.
